# Domoic acid biosynthesis and genome expansion in *Nitzschia navis-varingica*

**DOI:** 10.1128/mbio.02079-25

**Published:** 2025-10-30

**Authors:** Steffaney M. Wood-Rocca, Nicholas Allsing, Yasuhiro Ashida, Masaki Mochizuki, Malia L. Moore, Zoltán Füssy, Yuichi Kotaki, Clyde Puilingi, Yukari Maeno, Aodhan W. Beattie, Andrew E. Allen, Mari Yotsu-Yamashita, Todd P. Michael, Bradley S. Moore

**Affiliations:** 1 Center for Marine Biotechnology and Biomedicine, Scripps Institution of Oceanography, University of California San Diego, La Jolla, California, USA; 2 Environmental Genomics group, J. Craig Venter Institute, La Jolla, California, USA; 3 The Plant Molecular and Cellular Biology Laboratory, Salk Institute for Biological Sciences, La Jolla, California, USA; 4 Graduate School of Agricultural Science, Tohoku University, Aramaki-Aza-Aoba, Aoba-ku, Sendai, Japan; 5 Integrative Oceanography, Scripps Institution of Oceanography, University of California San Diego, La Jolla, California, USA; 6 Faculty of Science and Technology, Solomon Islands National University 228818, Honiara, Solomon Islands; 7 School of Science & Technology, Pacific Adventist University 140991, Port Moresby, Papua New Guinea; 8 Graduate School of Agricultural and Life Sciences, The University of Tokyo 515734 , Bunkyo-ku, Tokyo, Japan; 9 Skaggs School of Pharmacy and Pharmaceutical Sciences, University of California San Diego, La Jolla, California, USA; Massachusetts Institute of Technology, Cambridge, Massachusetts, USA

**Keywords:** diatom, genomes, enzymology, marine toxin, biosynthesis

## Abstract

Production of the neurotoxin domoic acid (DA) by benthic diatom *Nitzschia navis-varingica* poses considerable health and economic concerns. In this study, we employed whole genome sequencing and transcriptomic analyses of regionally distinct *N. navis-varingica* strains to unravel the genomic underpinnings of DA biosynthesis. Our analyses revealed sizable genomes—characterized by an abundance of repetitive elements and noncoding DNA—that exceed the size of any other pennate diatoms. Central to our findings is the discovery of an expanded domoic acid biosynthesis (*dab*) gene cluster, spanning over 60 kb and marked by a unique organization that includes core genes interspersed with additional genetic elements. Phylogenetic and syntenic comparisons indicate that transposition events may have driven the expansion and reorganization of this cluster. Biochemical assays validated that the kainoid synthase encoded by *dabC* catalyzes the formation of isodomoic acid B, thereby establishing a distinct chemotype in contrast to the DA profiles of planktonic diatoms. These results highlight the evolutionary trajectory of DA biosynthesis in diatoms and potential advantages conferred by genome expansion and enzyme diversification in dynamic marine environments.

**IMPORTANCE:**

Domoic acid (DA) is a potent neurotoxin produced by marine micro- and macroalgae problematic to fisheries and toxic to humans and animals. Our study elucidates the molecular mechanisms underlying DA production in the widespread Western Pacific benthic diatom, *Nitzschia navis-varingica*. Genomic and biochemical insights add information to our understanding of the evolution of toxin production across diverse phyla and also fill a gap in the knowledge of secondary metabolism in marine diatoms. These findings provide a genetic framework for identifying toxin production and its impacts in the benthos of vulnerable, coastal ecosystems.

## INTRODUCTION

**Fig 1:**
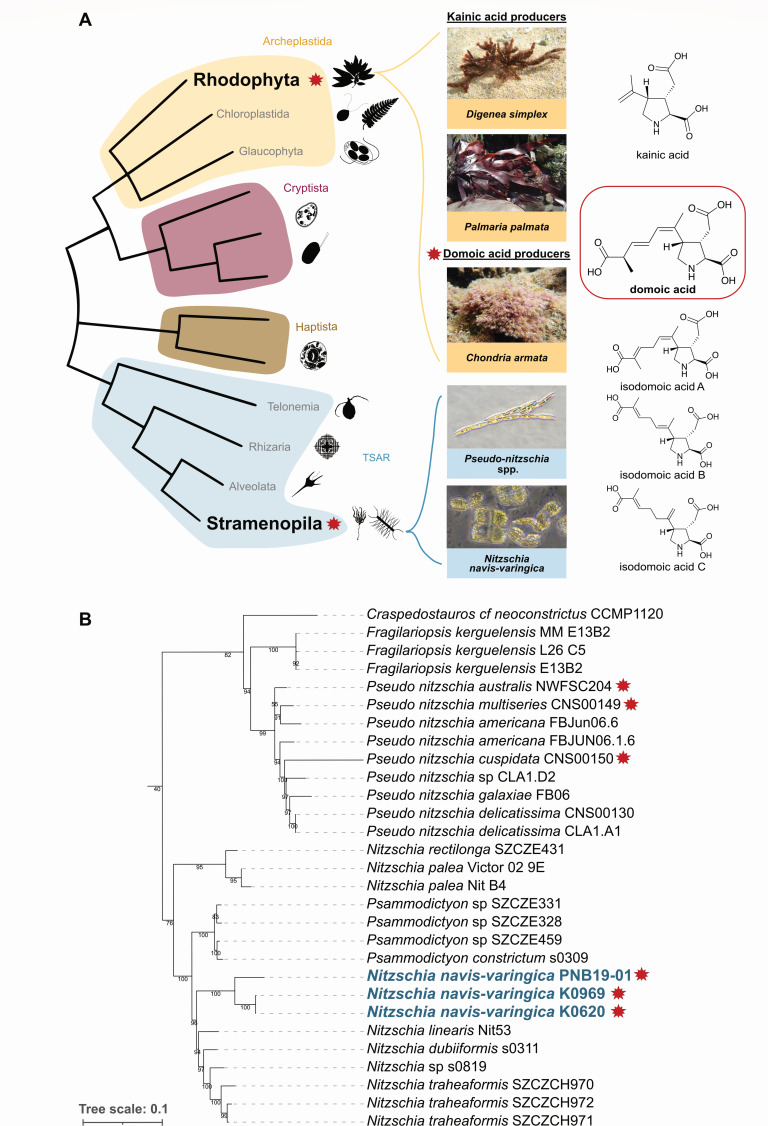
Kainoid production across marine eukaryotes. (**A**) Simplified eukaryotic tree of life based on Burki et al., with DA and kainic acid producers highlighted within their respective supergroups (8). (**B**) Rooted multi-locus phylogenetic analysis of alignments of 18S (SSU) and 23S (LSU) nuclear RNA genes as well as chloroplast genes *psbC* and *rbcL* from the Bacillariaceae family. Figure S1 shows a complete version of this phylogeny. Stars indicate *dab* clusters identified from this species. Bold indicates strains highlighted in this study. Images of *Chondria armata* (G. Saunders, University of New Brunswick) and *Digenia simplex* (T. Teruya, University of Ryukyus) are duplicated from our previous publication (9), reproduced here for context and comparison.

Domoic acid (DA) is a member of the kainoid class of natural neurotoxins, which includes the isomers and derivatives of DA, the namesake kainic acid, and acromelic acid (1, 2). Kainoids are non-proteinogenic amino acids characterized by a glutamate-derived pyrrolidine ring and multiple carboxylates (Fig. 1A). DA functions as a potent glutamate receptor agonist, leading to neurotoxic effects such as memory loss, disorientation, vomiting, seizures, and, in severe cases, coma and death (3–5). The first recorded outbreak of DA poisoning in humans, termed amnesic shellfish poisoning, occurred in 1987 in Prince Edward Island, Canada. This outbreak was attributed to the accumulation of DA in mussels that had consumed the toxin-producing diatom from what is now known as *Pseudo-nitzschia multiseries* (6, 7).

Since this deadly initial outbreak, subsequent research has described DA production in a variety of marine eukaryotic algae (10–13). Approximately half of the over 50 described species of harmful algal bloom-forming diatoms in the genus *Pseudo-nitzschia* have been reported to produce DA (14). These toxic blooms have caused significant negative economic impact and safety risks to humans and animals through bioaccumulation of DA in the marine food web (14–16). Additionally, red macroalgae of the family Rhodomelaceae—most notably *Chondria armata*, from which DA was first described—and other pennate diatoms such as *Amphora* sp., *Nitzschia bizertensis*, and *Nitzschia navis-varingica* have also been reported to produce this neurotoxin (9, 11, 14).

*N. navis-varingica* is primarily found in benthic ecosystems in the Western Pacific, where its DA production poses a threat to aquaculture facilities and shellfish harvested from mangrove ecosystems (17, 18). Although it has been isolated from the water column (19), *N. navis-varingica* is considered a benthic diatom due to its predominant occurrence in the benthos of intertidal environments, weakly adhering to mangrove roots and laboratory culture flasks alike (18, 20, 21). DA production in *N. navis-varingica* is widespread, corresponding with its ability to inhabit diverse ecological niches in conditions ranging from planktonic to benthic and euhaline to brackish systems (19, 21, 22). *N. navis-varingica* strains tend to produce higher ratios of isodomoic acid B to DA, in contrast to *Pseudo-nitzschia* spp. that primarily produce DA and isodomoic acid A (22, 23). Recent studies have demonstrated that geographically distinct populations display a high degree of genetic homogeneity, and strains with similar DA profiles are typically monophyletic, underscoring a genetic determinant in toxin production (18, 21, 24).

With the expansion of whole genomic sequencing of DA-producing algae, the evolution of DA biosynthesis is being unraveled (9, 25). DA biosynthesis genes (*dab*) were first identified in *P. multiseries* in a biosynthesis gene cluster (BGC) encoding an *N*-prenyltransferase (*dabA*), hypothetical protein (*dabB*), kainoid synthase (*dabC*), and cytochrome P450 (CYP450, *dabD*) (25). *Dab* gene clusters have thus far been identified in toxigenic *Pseudo-nitzschia* spp., including *P. multiseries*, *P. australis*, *P. seriata*, *P. cuspidata*, and *P. multistriata*, with their protein sequences displaying high amino acid identity (25–29).

DA and kainic acid biosynthesis have also been described in red macroalgae *Chondria armata* as well as *Digenia simplex* and *Palmaria palmata*, respectively (9, 30, 31). Despite the evolutionary distance between diatoms (Bacillariophyta) and red algae (Rhodophyta), the red algal domoic acid biosynthesis genes (*radACD*) display a high degree of gene synteny with both kainic acid biosynthesis genes (*kabAC*) and the DA biosynthesis (*dabABCD*) genes of *Pseudo-nitzschia* species (9). The unique structure and activity of these enzymes have led to additional research, as they represent a possible horizontal gene transfer event across distant taxa and novel biochemical mechanisms and enzyme structures (32–35). These discoveries highlight the complex evolutionary history of DA biosynthesis and underscore the need for further research.

Despite the incredible diversity within *Nitzschia*, comprising over 170,000 species and intraspecific names, only a handful of non-DA producers have been sequenced (36–38). Genomic studies of benthic diatoms such as *Nitzschia inconspicua* and *Seminavis robusta* revealed expansion of repetitive elements and unique adaptations to the benthos (37, 39). We therefore suspect *N. navis-varingica* will show a similar structure in line with its benthic adaptations. Moreover, these genomic data are essential for characterizing the evolution of the *dab* pathway in a novel lineage of algae.

To further investigate the evolution of DA biosynthesis and the ecological adaptations of sub/tropical benthic diatoms, herein we sequenced regionally distinct strains of *N. navis-varingica* for comparative genome and transcriptome analyses and DA biochemical validation. We report here that genome expansion in *N. navis-varingica* is mirrored in the enlarged organization of the *dab* BGC, which encodes the synthesis of the intermediate isodomoic acid B, in contrast to *Pseudo-nitzschia* diatoms, thereby suggesting a distinct evolutionary history in these diatom genera.

## RESULTS

### *Nitzschia navis-varingica* molecular taxonomy and genome characteristics

*N. navis-varingica* strains K0620 and K0969 were isolated from a marine shrimp culture pond and coastal waters in northern Vietnam, respectively (40). Strain PNB19-01 originated from Bootless Bay near Port Moresby, Papua New Guinea, following previous reports of DA and isodomoic acid B production (21). We investigated their molecular taxonomy within the Bacillariaceae family using a multi-locus phylogeny (18S rRNA, 23S rRNA, *psbC*, and *rbcL*). All three strains formed a well-supported, monophyletic lineage within a clade containing *Nitzschia linearis*, *Nitzschia dubiformis*, and *Nitzschia traheaformis*, remaining distinct from the DA-producing *Pseudo-nitzschia* clade (Fig. 1B). The *N. navis-varingica* clade is most closely related to *Psammodictyon* species, indicating that *N. navis-varingica* belongs to a separate lineage outside the major *Nitzschia* clades. The *Nitzschia* genus in this phylogeny is polyphyletic (Fig. S1), as has been previously reported and further evidenced by the clustering of *Nitzschia* spp. and *Psammodictyon*. Indeed, Mann et al. suggest that the two clades harboring *N. navis-varingica* may merit recognition as new genera, given the lack of clear morphological synapomorphies to unite them under *Nitzschia* as currently defined (36).

We produced partially phased genome assemblies of K0620 and K0969 and transcripts for PNB19-01 to investigate diversity in *dab* genes across DA-producing organisms as well as regionally distinct *N. navis-varingica* isolates. Genome and transcriptome sequencing for K0620 and K0969 was conducted using Pacific Biosciences (PacBio) long-read Single molecule real-time (SMRT) sequencing, while Illumina short-read RNA-seq was used to generate transcriptome data for PNB19-01. PacBio HiFi sequencing yielded approximately 35× coverage for K0620 (read N50 = 16,137 bp) and 31× coverage for K0969 (read N50 = 13,179 bp). IsoSeq transcriptome sequencing produced 13,042,775 reads (N50 = 1,415 bp) for K0620 and 11,434,165 reads (N50 = 1,537 bp) for K0969. K-mer frequency analysis of the HiFi reads revealed distinct homozygous and heterozygous peaks, along with elevated duplication signals, consistent with a diploid genome architecture and a pronounced lower-coverage heterozygous peak (Fig. S2). For PNB19-01, Illumina RNA-seq performed by Novogene yielded 12,640,360 reads totaling 3.79 Gbp, with a read N50 of 1,759 bp.

The PacBio HiFi reads for both K0620 and K0969 were assembled into haplotype-resolved genome assemblies. Although both haplotypes were retained, haplotype 1 (hap1) was selected for downstream analyses due to its higher overall quality (Table S1). As part of the assembly pipeline, contigs were also screened for contamination using the National Center for Biotechnology (NCBI) Foreign Contamination Screening-GX (FCS-GX) (41). The FCS-GX pipeline removed 66.3 and 121.4 Mbp potential contaminants from K0620 and K0969, respectively, resulting in hap1 assembly sizes of 879.5 Mbp for K0620 (N50 = 303,884 bp) and 782.8 Mbp for K0969 (N50 = 134,724 bp) (Table S2). However, evaluating the GC content of the remaining contigs suggested that a residual amount of bacterial contamination remained. Therefore, we further filtered the contigs using Blobtools, and an additional 17.2 and 16.3 Mbp were removed from K0620 and K0969, respectively (Fig. S3; Table S2). The resulting assemblies were high quality with BUSCO (Stramenopiles) completeness scores of 94% and 85% for K0620 and K0969, respectively (Table 1). BUSCO analysis also revealed duplicated gene content of 19% and 31%, respectively, consistent with retention of allelic variants in the partially phased assemblies. Supporting transcriptome completeness, IsoSeq mapping showed that 91.5% of reads from K0620 and 83% from K0969 aligned to their respective assemblies.

**TABLE 1:** Genome characteristics of select pennate diatoms^*a*^^,^^*b*^

Parameter	*Nitzschia navis-varingica* K0620	*Nitzschia navis-varingica* K0969	*Nitzschia inconspicua* strain hildebrandi GAI-293	*Pseudo-nitzschia multistriata* B856	*Pseudo-nitzschia multiseries* CNS08	*Pseudo-nitzschia delicatissima* CNS07	*Fragilariopsis cylindrus* CCMP1102	*Fistulifera solaris* JPCC DA0580	*Seminavis robusta* D6
Haploid genome size (Mbp)	862.3	766.5	49.9	56.8	252.4	34.1	80.5	49.7	125.6
N50 (Mbp)	0.306	0.136	3.7	0.14	0.80	2.90	0.78	0.33	0.05
BUSCO completeness (%)	94	85	100	86	81.5	73.9	95	97	99
Predicted proteins	46,987	41,796	17,968	12,039	18,649	14,375	18,111	20,429	36,254
Repetitive elements in genome (%)	71	70	41	–	68.7	1.73	–	16	22.64
Sequencing technology	PacBio	PacBio	PacBio	Sanger	PacBio, Hi-C	PacBio, Hi-C	Sanger	454	PacBio, Illumina
Reference	This study	This study	Oliver et al. (37)	Russo et al. (42)	He et al. (26)	He et al. (26)	Mock et al. (43)	Tanaka et al. (44)	Osuna-Cruz et al. (39)

^*a*^ See Table S1 for complete statistics for genomes produced in this study.

^*b*^ “–” indicates data not available.

**Fig 2:**
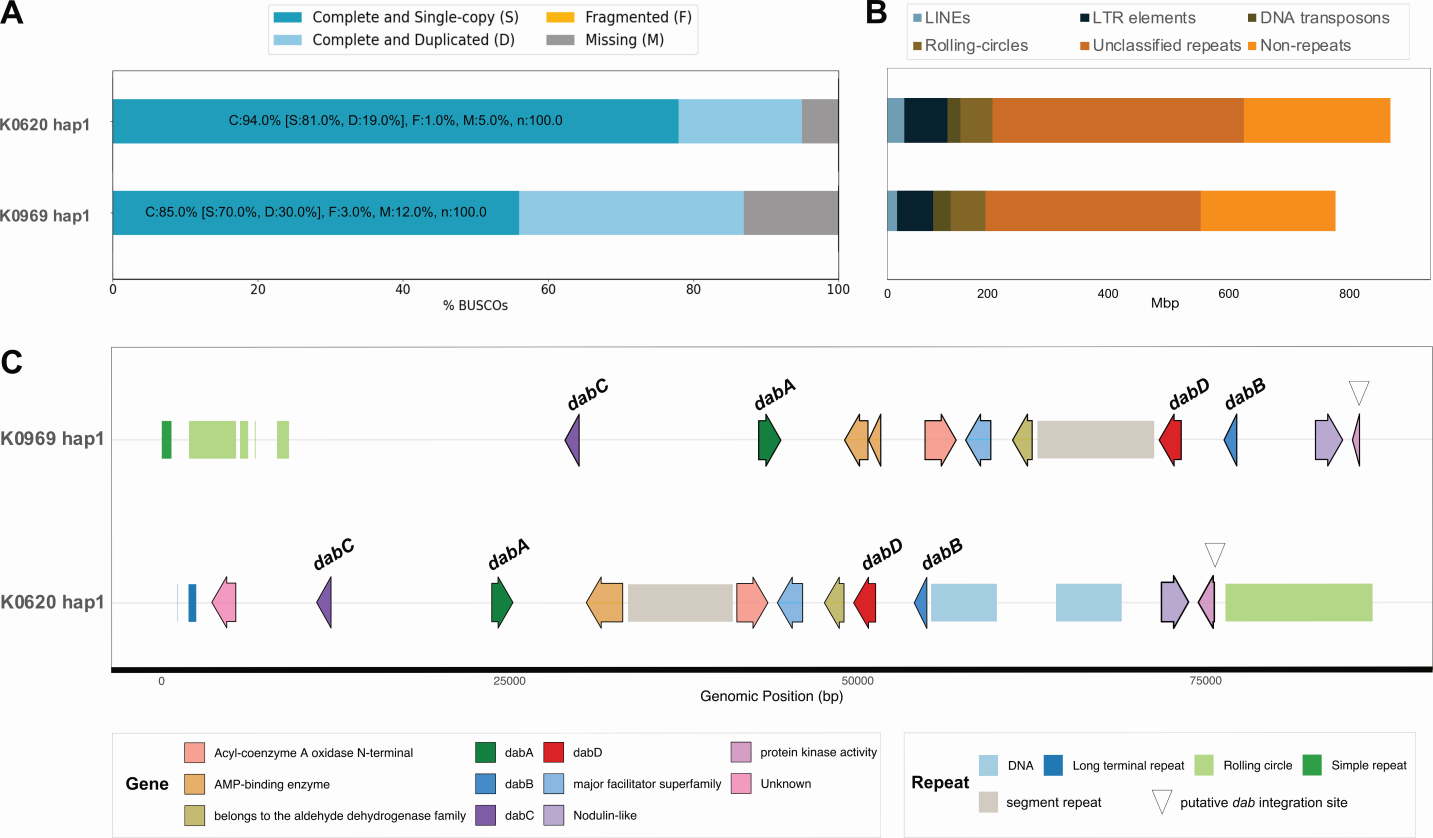
Genome assembly quality and repeat composition across haplotypes. (**A**) BUSCO completeness assessments highlighting the proportion of complete single-copy (S), duplicated (D), fragmented (F), and missing (M) orthologs in each assembly. (**B**) Length of different repeat elements within genome assemblies of K0969 and K0620. Categories include long interspersed nuclear elements (LINEs), LTR elements, DNA transposons, rolling circles, unclassified repeats, and non-repeats. See haplotype genome and repeat statistics in Table S1. (**C**) Genomic neighborhood of the *dab* gene cluster. Arrows represent genes, and bars represent repetitive elements. See full-length *dab*-containing contigs in Fig. S6.

Both *N. navis-varingica* genomes are at least 15 times the size of *Nitzschia inconspicua* strain hildebrandi GAI-293 and up to 22 times the size of other pennate diatoms such as *Pseudo-nitzschia delicatissima*. The genome size in *N. navis-varingica* is shaped by the large proportion of non-protein-coding regions and repetitive DNA (Fig. 2B). Both strains constitute only approximately 7% coding sequences, in contrast to approximately 70% repetitive DNA. Despite this, they still contain more predicted proteins than other pennate diatom genomes (Table 1). Of the various classes of repetitive DNA, unclassified repetitive elements make up the largest fraction of the genomes (48%–50%), followed by long-terminal repeats (LTR) (8%–9%) and rolling circles (6%–8%). A similar makeup has been previously reported in centric diatom genomes (45).

Of the functionally annotated genes, the majority correspond to core metabolic processes (Fig. S4). The prevalence of annotations related to macromolecule transport, such as bicarbonate, nitrate, and magnesium, light harvesting, and stress response possibly reflects adaptations to the environmental fluctuation of pH, light and nutrient availability, and temperature that are common in the sub/tropical benthos (46–48). Given the ability of benthic *Nitzschia* spp. to produce volatile, halogenated hydrocarbons, like methyl iodide (49–51), we queried the genomes for putative haloperoxidase and halide methyltransferase genes. We observed an interesting co-localization of genes encoding a putative halide methyltransferase with an iodotyrosine deiodinase, the latter of which had homologs in benthic diatom *Seminavis robusta* and various Alveolates (Table S3; Fig. S5). The *N. navis-varingica* iodotyrosine deiodinase may function to detoxify halogenated amino acids, which in turn would result in cellular iodide that would be methylated and removed as volatile methyl iodide (51–55).

### Domoic acid biosynthesis gene cluster identification

We identified the *dab* gene cluster in the genomes of *N. navis-varingica* K0620 and K0969 using BLAST and HMM searches with the existing collection of *Pseudo-nitzschia dab* genes and *C. armata* red algal domoic acid (*rad*) genes. Initial searches revealed the co-localization of homologs for *dabA*, *dabB*, *dabC*, and a CYP450 unrelated to canonical *dabD* genes in both K0620 and K0969 (Fig. 2; Fig. S6). An additional copy of *dabC* was also identified in K0620 (referred to as *dabC2*) on a separate contig (Fig. S7). Notably, we identified a similar set of *dabABC* and a CYP450 transcript in *N. navis-varingica* PNB19-01. Curiously, the CYP450 sequences that displayed the strongest homology with *Pseudo-nitzschia dabD* sequences were not co-localized with *dabABC* in K0620 and K0969. Rather, the co-localized CYP450s only exhibited up to 21% amino acid identity with known *dabD* sequences, a trend also observed in *C. armata* (9) (Fig. S8). We thus suspect that the co-clustered CYP450, which is conserved across all three strains, functions as the oxygenase DabD. Additionally, the *N. navis-varingica dab* cluster contains the *dabB* gene, which encodes a protein of unknown function as first reported in *Pseudo-nitzschia* diatoms, yet not in DA-producing red algae (25).

The *N. navis-varingica dab* cluster spans over 60 kb, features a unique gene arrangement, and contains additional genes and repetitive elements (Fig. 2C). The expansion of intergenic space and the abundance of repeats at the *dab* locus mirror the overall genome size and composition. The cluster is flanked by DNA transposons and rolling circle elements, suggesting that transposition events may have contributed to its expansion and rearrangement. Within the cluster, interspersed genes encode proteins with domains such as aldehyde dehydrogenase, major facilitator superfamily, acyl-coenzyme A oxidase, and AMP-binding enzymes. To further determine if these genes were potential misannotated repetitive elements, the intergenic space between *dabA* and *dabD* was BLAST searched against the remainder of the genome assembly (56). Results showed that portions of the noncoding intergenic space, but not the inserted coding sequences, displayed high (up to 95%) nucleotide similarity throughout the genome (Fig. S9).

The gene order of the *N. navis-varingica dab* cluster is rearranged relative to other DA-producing organisms: *dabA* and *dabC* are adjacent, followed by a series of interspersed genes, then *dabB* and the putative *dabD*. The CoA-binding protein and protein kinase genes that flank a block containing the aldehyde dehydrogenase, major facilitator superfamily member, *dabB*, *dabD*, and a nodulin-like gene are similar to the sequences found in other diatom genomes.

The putative integration site for the *Pseudo-nitzschia dab* gene cluster, involving a highly conserved CoA-binding protein (K0620 hap1 g25680) and protein kinase (K0620 hap1 g25681) gene pair as proposed by He et al., is present in *N. navis-varingica* in a genomic region separate from the *dab* cluster (26). Interestingly, genes containing similar domains are found in the *N. navis-varingica dab* locus. The protein kinase gene found downstream of the *N. navis-varingica dab* cluster is more homologous (31% amino acid identity) to the sequence found in putative *Pseudo-nitzschia dab* insertion site than the CoA-binding gene (19% amino acid identity). Phylogenetic analysis of these sequences shows that these sequences are highly conserved across diatoms and other stramenopiles (Fig. S10). Additionally, sequence motif enrichment analysis of the putative integration site reveals highly conserved diatom basic leucine zipper (bZIP) transcription factor binding motifs, which are abundant in diatoms and other heterokonts (Fig. S11) (57). These observations suggest that the putative integration site may be highly conserved across many diatom lineages. The presence of an orthologous protein kinase may indicate a possible conserved mechanism of cluster integration across these taxa.

### *N. navis-varingica* compound detection and biochemical validation of isodomoic acid B synthase

**Fig 3:**
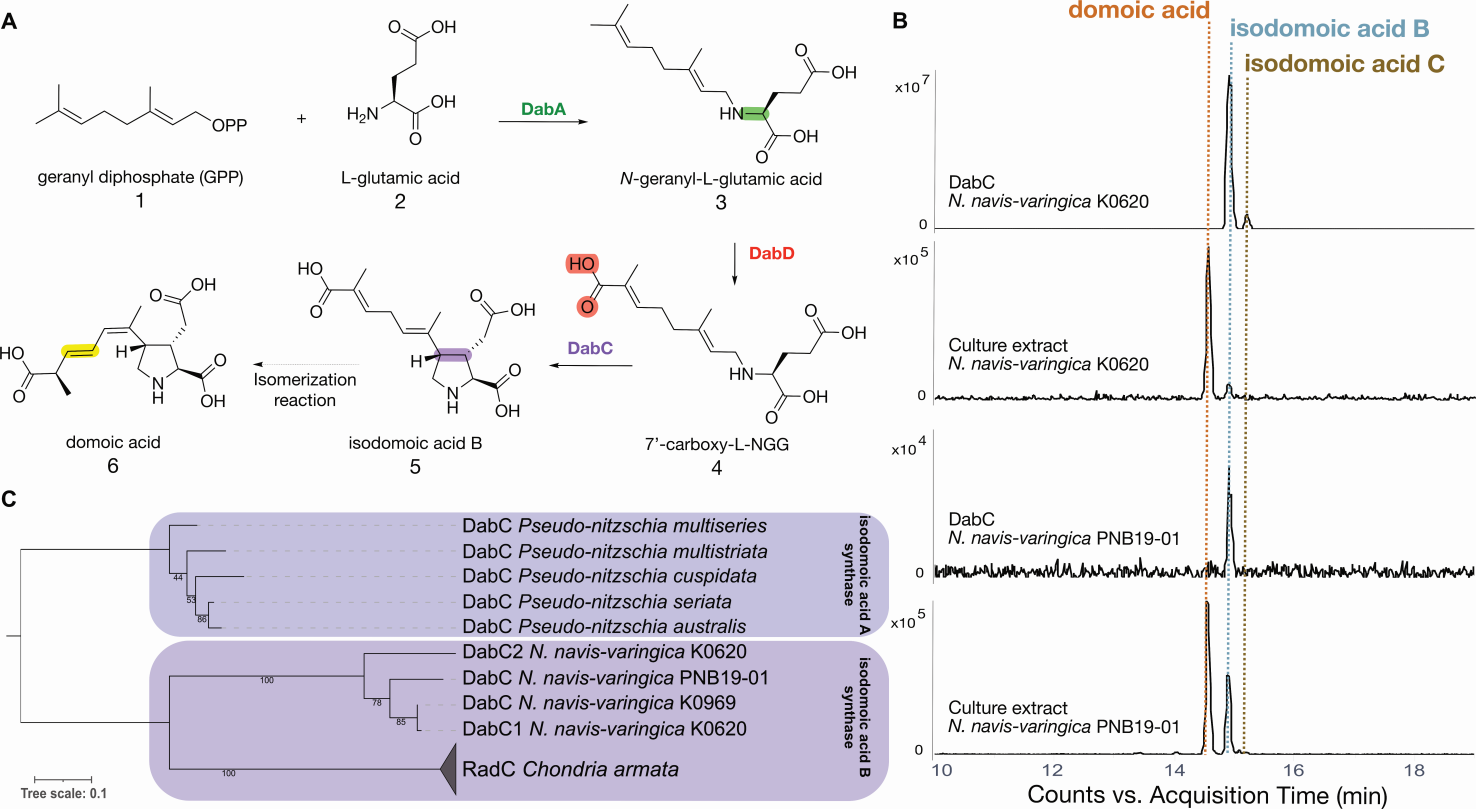
Domoic acid biosynthesis pathway in *N. navis-varingica* proceeds through isodomoic acid B. (**A**) *N. navis-varingica* Dab enzymes collectively convert L-glutamic acid (L-Glu) and geranyl diphosphate (GPP) to isodomoic acid B on route to DA. (**B**) Extracted ion chromatogram profiles for DA and isodomoic acid B (*m/z* 312.1 ± 1.0) from DabC assays and culture extracts. See comparison to synthetic standards in Fig. S12. (**C**) Branching of isodomic acid B synthase DabC amino acid sequences visible through unrooted phylogeny of kainoid synthase enzymes. Clade shading indicates isodomoic acid A or B production.

Analysis of *N. navis-varingica* culture extracts using liquid chromatography-mass spectrometry (LC-MS) revealed the presence of DA along with related isomers isodomoic acid A and isodomoic acid B (Fig. 3; Fig. S12). While *N. navis-varingica* cultures primarily produce DA, isodomoic acid B is present at substantial levels—a pattern that contrasts with DA-producing *Pseudo-nitzschia* species, which predominantly yield DA and isodomoic acid A. Based on these observations, we hypothesized that the kainoid synthase enzyme (DabC) in *N. navis-varingica* generates isodomoic acid B, a pattern found in the isodomoic acid B chemotype seen in *C. armata* (9).

To validate this hypothesis, we examined key steps in the DA biosynthesis pathway. Enzyme assays with purified glutamate *N*-prenyltransferase enzymes (K0620-NnvDabA and PNB19-01-NnvDabA) confirmed the production of *N*-geranyl-L-glutamic acid (Fig. 3, compound **3**, L-NGG) from geranyl diphosphate (**1**) and L-glutamate (**2**; Fig. S13). Subsequent assays of the kainoid synthase enzymes K0620-NnvDabC and PNB19-01-NnvDabC revealed that they catalyzed the oxidative cyclization of linear precursor 7´-carboxy-L-NGG (**4**), yielding isodomoic acid B (**5**) as the product (Fig. 3B). These results establish that *N. navis-varingica* exhibits a distinct chemotype from other DA-producing diatoms in its production of isodomoic acid B. It also underscores the role of the kainoid synthase in driving isomer diversity within the DA biosynthesis pathway.

NnvDabC enzymes also cyclize L-NGG (**3**) to produce primarily dainic acid A (Fig. S14). NnvDabC from strain K0620 also appears to produce trace amounts of dainic acid B/C, which have been shown to co-elute using our methods (9, 25). This is consistent with enzymatic products produced by isodomoic acid B synthase RadC and *P. multiseries* isodomoic acid A synthase DabC, but a marked discrepancy from KabC, which cannot cyclize the geranyl side chain present in *dab* pathway intermediates (16). It should also be noted that dainic acid A can sometimes be detected in culture extracts of *N. navis-varingica* (Fig. S15), as has been previously reported in *C. armata* (31).

### Gene synteny and phylogenetic analysis of kainoid biosynthesis gene clusters

Phylogenetic analysis of individual *dab* genes largely aligns with previous studies (9, 25, 30, 32). Isodomoic acid B synthase NnvDabC groups more closely with RadC, rather than with the isodomoic acid A synthases found in related *Pseudo-nitzschia* species (Fig. 3C). All kainoid synthase DabC enzymes nonetheless form a subclade in the broader phylogeny of bacterial and fungal alpha-ketoglutarate-dependent Fe(II)-containing dioxygenases, as previously observed (9), highlighting the sources of a likely horizontal gene transfer event (Fig. S16A). DabB phylogeny shows that NnvDabB groups separately from *Pseudo-nitzschia* spp. DabB sequences, and other uncharacterized protein sequences in the tree are distantly related (Fig. S17). *In silico* analysis of NnvDabB indicates that it contains an N-terminal signal peptide and may act as a type II signal anchor that anchors proteins to the membrane (58, 59).

Broader phylogeny of DabD sequences within CYP450s highlights the uniqueness of *N. navis-varingica* DabD, which groups with other diatom CYP450 sequences but separately from *Pseudo-nitzschia* spp. DabD, also echoing the possibility of gene duplication and lineage-specific neofunctionalization of CYP450 enzymes as reported in the *rad* pathway (9) (Fig. S16B). To understand the evolutionary origin of the taxonomically distinct NnvDabD co-localized with the *dab* cluster, we constructed a phylogenetic tree of all 128 annotated K0620 CYP450 sequences (Fig. S18). While the *dabD* gene candidate predicted by homology to the characterized *Pseudo-nitzschia* spp. *dabD* formed a clade with CYPs of mostly unknown function, the *dabD* candidate from the BGC groups with CYP450s widely annotated as retinoid hydroxylases. This result indicates a possible origin of the candidate CYP450 DabD in carotenoid biosynthesis, potentially resulting from similarities in structural motifs of the two isoprenoid species.

To gain further insights into the kainoid synthase DabC and CYP450 DabD enzymes, we generated AlphaFold2 structural models and CASTp 3.0 binding pocket area and volume models (60, 61) (Fig. S19). All AlphaFold2 models reached high confidence (pTM ≥0.85). Similarity of the structures reflects their sequence phylogeny. Sequence-structure matchmaker alignment of *P. multiseries* DabC and DabD to their *C. armata* (alignment score 1,219.2, pruned root-mean-square deviations [RMSD] 0.665 Å) and *N. navis-varingica* (score 1,239.1, pruned RMSD 0.666 Å) homologs reveals high structural similarity among the kainoid synthase enzymes, whereas PmDabD aligned more closely to RadD (score 662.9, pruned RMSD 1.124 Å) than NnvDabD (614.4, pruned RMSD 1.198 Å) (62). Interestingly, NnvDabC1 modeled pocket volume (360.6 Å^3^) is approximately twice that of PmDabC (162.1 Å^3^) and RadC (139.6 Å^3^) (Fig. S19). Large, modeled pocket volumes (>1,300 Å^3^) of D-type enzymes reflect those of other flexible CYP450 binding pockets (62).

**Fig 4:**
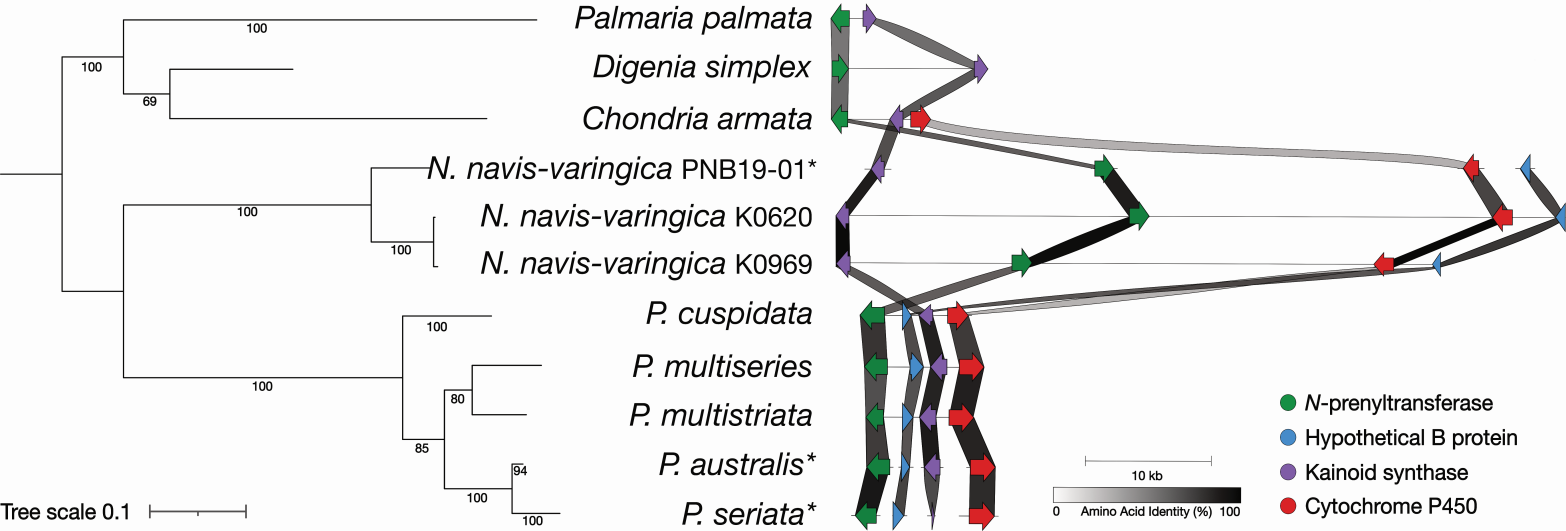
Syntenic comparisons and phylogenetic analysis of kainoid biosynthesis gene clusters. A phylogram represents unrooted phylogeny of concatenated kainoid biosynthesis genes coupled with visualization of the corresponding biosynthesis gene clusters. Lines represent contiguous stretches of DNA; arrows represent genes and their orientation; shading between genes represents amino acid sequence identity. *Transcripts.

In the larger context of kainoid BGCs, a concatenated phylogeny of *N*-prenyltransferase (A-type), B proteins (unknown function), kainoid synthase (C-type), and CYP450 (D-type) genes forms a well-supported *N. navis-varingica* clade distinct from both the *Pseudo-nitzschia dab* clusters and the red algal (Rhodophyta) domoic/kainic acid pathways (Fig. 4). Notably, gene synteny for *dabA* and *dabC* is highly conserved among *N. navis-varingica* strains, with amino acid identities ranging from 84% to 99%. These findings underscore a unique evolutionary route for isodomoic acid B biosynthesis in *N. navis-varingica* and highlight the potential for divergent enzymatic adaptations in diatom versus red algal kainoid production.

## DISCUSSION

This study describes the DA biosynthesis pathway in pennate, benthic diatom *N. navis-varingica*. By combining genomic and transcriptomic analysis of regionally distinct strains of *N. navis-varingica*, we uncovered a novel organization of the *dab* gene cluster, assembled two high-quality diatom genomes, and clarified the phylogenetic position of *N. navis-varingica* within Bacillariaceae. Additionally, *in vitro* biochemical assays with key biosynthesis enzymes validated the DA isomer profile across strains, leading to the discovery of a diatom isodomoic acid B synthase, NnvDabC, which represents a novel enzymatic function within DA-producing diatoms.

### Gene cluster organization and evolution

The *dab* gene cluster in *N. navis-varingica* strains uniquely spans over 60 kb, which is roughly 10 times larger than *dab* clusters found in *Pseudo-nitzschia* spp. and *C. armata* (9, 25). Expansion of BGCs through duplication and recruitment of additional enzymes has often been cited in eukaryotes for the diversification of secondary metabolite production (63–67). To our knowledge, this is the first instance of secondary metabolite BGC expansion in diatoms. Identification of an expanded BGC highlights the power of long-read sequencing. During our review process, a recent paper reported *dab* genes in draft Illumina assemblies of several *N. navis-varingica* strains, but they were fragmented across separate contigs (68). The *dab* cluster expansion is further supported by an additional copy of *dabC* outside of the cluster, the co-localization of *dab* genes with repetitive elements, and interspersion of additional genes. Based on the genomic arrangement and gene synteny conservation among both *N. navis-varingica* strains and across diverse taxa, we hypothesize that core kainoid biosynthesis genes *dabA* and *dabC* form a single module within the cluster. The presence of flanking DNA transposons also supports the current proposal of DA evolution, in which horizontal gene transfer contributed to the acquisition of this module. This observation is consistent with reports that diatoms may have acquired up to 5% of their genes through horizontal gene transfer (69). The downstream presence of *dabB* and *dabD* represents an additional module that was likely recruited through neofunctionalization and intra-genomic reorganization to confer the production of DA. The repetitive segment between *dabA* and *dabD*, displaying over 90% nucleotide identity in approximately 100 other places in the genome, further supports this hypothesis. Notably, the protein of unknown function *dabB* is consistently found in diatom *dab* clusters but is absent in the red alga *C. armata*, suggesting a lineage-specific recruitment event that may contribute to differences in DA biosynthesis between these groups. Phylogenetic analysis of hypothetically assigned NnvDabD suggests its recruitment via neofunctionalization of native biochemistry, as was found in *C. armata* (9).

The conservation of a protein kinase downstream of the *dabBD* module—identified as a potential *dab* cluster integration site in *P. cuspidata*—suggests that this region may favor DA biosynthesis insertion and regulation (26). Phylogenetic analysis shows that the protein kinase is orthologous to sequences found in *P. multiseries* and *P. multistriata*, indicating that this locus may favor *dab* cluster insertion or facilitate genomic rearrangements involving the putative neofunctionalized CYP450. However, the CoA-binding gene present at the *dab* locus is not orthologous to sequences found in *Pseudo-nitzschia*; instead, the orthologous CoA-binding and protein kinase gene pair exists outside the cluster in *N. navis-varingica*. This implies that their co-localization with the *dab* cluster may be coincidental rather than representing a conserved regulatory or integration hotspot.

This regulatory context is particularly relevant when considering the evolution of the cluster toward DA production, which implies that DA confers ecological advantages, such as in response to pH or grazer stress (25, 28, 70–74). For example, the regulation of putative CYP450 DabD modulates the addition of the carboxylic acid moiety crucial for DA’s toxicity, supporting its potential role in deterring grazers (33). Indeed, the *N. navis-varingica* K0620 strain sequenced in this study was shown to deter grazing by the mixotrophic dinoflagellate *Karlodinium armiger* (75). Additionally, DA production may be linked to pH tolerance given that *N. navis-varingica* is found in shallow, brackish environments with elevated pH, whereas DA production in *Pseudo-nitzschia* is linked with elevated pCO_2_ (25, 74, 76, 77). Further investigation of the regulatory network modulating DA production in response to environmental stress should be done by investigating the conservation of *dab* gene expression patterns in *N. navis-varingica* and DA-producing *Pseudo-nitzschia* species.

### Mechanistic insights and enzymatic functions

Our work also sheds light on the evolution of the gene cluster toward DA production through functional diversification of kainoid synthase enzymes. Our biochemically supported phylogenetic analysis shows that isodomoic acid B synthase NnvDabC1 groups functionally with RadC, with both enzymes converting L-NGG (**3**) and cNGG (**4**) to DA-like molecules dainic acid A and isodomoic acid B (**5**), respectively. DA-producer *C. armata* RadC behaves similarly, whereas kainic acid-producing *D. simplex* KabC does not (9, 30). *P. multiseries* DabC was demonstrated to act as an isodomoic acid A synthase, whereas we have shown that *N. navis-varingica* DabC functions as an isodomoic acid B synthase, demonstrating a distinct evolutionary trajectory between these genera.

The function of NnvDabC1, the presence of additional genes at the *dab* locus, *dabC* paralogs outside of the *dab* cluster, and the low homology of putative CYP450 NnvDabD raise important biosynthesis questions about the order of operations in the pathway as well as the elusive final step—namely, the conversion of isodomoic acid A or B to DA. Conversion of isodomoic acid B to DA not only requires 1,3-olefin migration but also an additional and separate *trans*-to*-cis* isomerization of the second olefin. Sequence and structural analysis reveals that CYP450 NnvDabD exhibits significantly lower homology to other DabD enzymes in other *dab* and *rad* clusters, suggesting it may catalyze a distinct reaction. Consequently, rather than performing the precedented carboxylation of L-NGG (**3**), the pathway may proceed through the formation of dainic acid intermediates. Indeed, the increased modeled binding pocket volume of NnvDabC1, in comparison with PmDabC and RadC, may allow for increased substrate flexibility and therefore both pathways to occur in *N. navis-varingica*. This possibility is strengthened by the presence of dainic acids in culture extracts.

The discovery of additional oxidative enzymes—beyond the core alpha-ketoglutarate-dependent Fe(II)-containing dioxygenase C protein and CYP450 D protein—such as genes encoding acyl-CoA oxidase and aldehyde dehydrogenase within the *dab* locus may imply involvement in double-bond isomerization. For example, these additional enzymes could induce oxidative rearrangement of the double bond to be shifted out of conjugation with the carboxylate or work in concert with core oxidative enzymes in the *dab* cluster (78–80). Alternatively, though less precedented, activation of isodomoic acids by an AMP-binding protein could allow for double-bond isomerization by a dehydrogenase enzyme, in a manner similar to bacillaene biosynthesis and other double-bond isomerization biosynthesis strategies present in polyketide systems (81–83). Although further work is required to fully elucidate the order and function of these steps, these potential mechanisms align with rare biosynthesis strategies in which oxidative enzyme-catalyzed saturation of unactivated double bonds results in olefin migration (79, 84, 85). However, the presence of *dabC* paralogs outside of the *dab* cluster implies that other genes outside the *dab* locus may be involved in this pathway as well.

### Ecological and evolutionary implications of genome expansion

Our study also found that *N. navis-varingic*a strains have exceptionally large genomes relative to other pennate and benthic diatoms. The over 750 Mbp genomes are more than 15 times the size of closely related *N. inconspicua* str. *hildebrandi,* but only six times the size of the benthic diatom *Seminavis robusta* (37, 39). Previous research with strain K0620 sequences measured large cell biovolume (3,500 µm^3^) and slow doubling rate (51.4 h), which are both correlated with larger genome size in diatoms (45, 75). Similar to the observations of the centric diatom *Thalassiosira* (with genomes up to 1.5 Gbp) and across eukaryotes, genome expansion in *N. navis-varingica* appears to be due to the expansion of noncoding DNA (45, 86, 87). This result is intriguing because Bergmann’s rule predicts smaller cells, and therefore genome sizes, at warmer temperatures, which are typical of sub/tropical benthic environments (88–90). Our findings add to emerging evidence that diatoms may defy this ecological principle, potentially contributing more to global primary production under the threat of rising ocean temperatures (91–93). In this instance, the size of the *N. navis-varingica* genome may correlate with increased genetic diversity and adaptations to fluctuating salinity, pH, light, and nutrient availability, which in turn could lead to greater cell abundance—a trend in support of the latter hypothesis and observed in polar diatom communities (45). Moreover, DA-producing species of *Pseudo-nitzschia* have augmented genomes with increased repetitive elements, relative to non-DA-producing species (26, 27). Genomes of non-DA-producing strains of *N. navis-varingica* may reveal a similar trend of differential DNA content.

### Taxonomic and phylogenomic considerations

Following the first outbreak of DA poisoning by the diatom formerly known as *Nitzschia f. pungens* (now *Pseudo-nitzschia multiseries*), the genus *Pseudo-nitzschia* was delineated from *Nitzschia* partly through taxonomic research driven by DA production (6, 7). Our work extends this re-evaluation to *N. navis-varingica*, another DA producer within the Bacilliaracae family, which has been documented as widespread throughout the Western Pacific and harbors significant intraspecific diversity (24, 36). Our analysis of multiple conserved genetic markers of the speciose *Nitzschia* genus and Bacillariaceae family reveals that *N. navis-varingica* strains clade with a subset of *Nitzschia* spp. and *Psammodictyon* spp. commonly found in the benthos of the western sub/tropical Pacific. Given its phylogenetic position outside the primary *Nitzschia* clades, the reported absence of a unifying synapomorphy, and the presence of DA-producing species, this “cryptic” clade is a strong candidate for taxonomic revision (36, 94). Further phylogenomic investigations are recommended to resolve these taxonomic ambiguities and elucidate the origins of DA production within the Bacillariaceae.

### Conclusion

In summary, our study provides new insights into the evolution and organization of the DA biosynthesis pathway by the identification of a novel *dab* cluster in *N. navis-varingica*. The unique genome-wide expansion reflects the expansion and modular reorganization of the *dab* gene cluster. The discovery of the genetic basis of DA production in *N. navis-varingica* and biochemical verification of the Dab pathway suggest that DA production may offer ecological advantages in the subtropical benthic habitat. Future research should explore *N. navis-varingica dab* gene expression patterns and taxonomic relationships among DA-producing diatoms to better understand the ecological and evolutionary implications of DA production.

## MATERIALS AND METHODS

### Diatom culturing and harvesting

*Nitzschia navis-varingica* strains K0620 and K0969 were obtained from the Norwegian Culture Collection of Algae (https://norcca.scrol.net/). Strains were maintained in natural seawater F/2 media (95), at 16°C, and under a 12:12 photoperiod. Strain K0969 was incidentally co-cultured with a *Pseudobodo* sp. (Bicoecea), present from the culture collection. Attempts to isolate strain K0969 without the contaminant were unsuccessful.

PNB19-01 (21) was cultured in 30 mL of F/2 medium in a 50 mL tissue culture flask (Greiner bio-one, Tokyo, Japan) by incubating it at 25°C under an irradiance level of 80 µmol photons m^−2^ s^−1^, with a 12:12 h light:dark cycle. The medium was prepared using seawater diluted with distilled water to a salinity of ca. 28.

For scale-up culturing, K0620 and K0969 were first treated with antibiotics in the following manner. Once cells reached the exponential phase, they were treated with antibiotics added sequentially over 3 consecutive days. On the 1st day, Provasoli’s concentrated antibiotic solution (Sigma P8029) was added at final concentrations of 480 units/mL penicillin G, 2 µg/mL chloramphenicol, 12 units/mL polymyxin B, and 2.4 µg/mL neomycin. On the 2nd day, carbenicillin (500 µg/mL final concentration) and cefotaxime (250 µg/mL final concentration) were added. On the 3rd day, a penicillin-streptomycin-neomycin mixture (Gibco) was added at final concentrations of 50 µg/mL penicillin, 50 µg/mL streptomycin, and 100 µg/mL neomycin. They were then grown at a 4 L scale at 20°C until exponential growth phase, approximately 1 week. To maintain the exponential phase, 2 L of cell culture was harvested by centrifugation at 7,000 × *g* for 20 min every 4–5 days. The remainder of the culture was replenished with 2 L F media. This was repeated until sufficient biomass was collected.

### DNA and RNA extraction

For strains K0620 and K0960, high molecular weight (HMW) DNA was extracted from 0.1 g biomass using Illustra Nucleon Phytopure Genomic DNA Extraction Kit (Cytiva). Manufacturer’s instructions were followed with the following exception: the chloroform extraction and DNA precipitation steps were repeated five times in order to increase the quality of DNA. Extracted DNA was size-selected using the BluePippin system with a High Pass Plus 15 kb cassette (Sage Science Cat# BPLUS03), and HMW fragment lengths were verified using the 4150 TapeStation (Agilent Cat# G2992AA) with a Genomic DNA ScreenTape (Agilent Cat# 5067-5365). RNA was extracted from 0.1 g of biomass using the Direct-zol RNA Purification Kit (Zymo) following the manufacturer’s instructions. RNA was quantified and quality assessed using a Qubit RNA BR Assay Kit (Invitrogen Cat# Q33231) and TapeStation RNA ScreenTape (Agilent Cat# 5067-5576).

For strain PNB19-01, the 29 day culture (15 mL) was harvested 3.3 h after switching the lighting from dark to light by centrifugation in a 50 mL conical tube at 670 *g* for 1 min at 25°C. After removal of the supernatant, TRI reagent (Sigma, Cat# T9424, 1 mL) and 0.1 mL of Zirconia Ball YTZ-0.05 mm (Nikkato Corporation, Japan) were added to the cells. Then the cells were disrupted using an MS-100 (TOMY, Japan) at 3,000 rpm for 1 min. The suspension was transferred into a microtube (1.5 mL), and chloroform (0.2 mL) was added, then kept for 5 min at room temperature (RT). The mixture was centrifuged for 15 min at 20,600 × *g* at 4°C. The supernatant was transferred to a new microtube, then isopropanol (0.5 mL) was added and kept for 5 min at RT. After centrifugation for 15 min at 20,600 × *g* at 4°C, the supernatant was removed, then 75% EtOH (1 mL) was added to the precipitate, then centrifuged again for 6 min at 5,000 *g* at 4°C. After removal of the supernatant, the precipitate was dissolved in RNase-free water (15 µL) and mixed with DNase I 10× buffer (1.5 µL) and DNase I (RNase-free, Nippon Gene, 0.5 µL) using vortex, then kept at 37°C for 10 min. After the reaction, TRI reagent (0.5 mL) and chloroform (0.1 mL) were added to the reactant and kept 5 min at RT, then centrifuged for 15 min at 20,600 × *g* at 4°C. The supernatant was transferred to a new microtube, then isopropanol (0.25 mL) was added. After keeping at RT for 5 min, the mixture was centrifuged for 15 min at 20,600 × *g* at 4°C. The supernatant was removed, then 75% EtOH (0.5 mL) was added, then centrifuged for 6 min at 5,000 × *g* at 4°C. The obtained total RNA was dissolved with 50 µL of RNase-free water, then quantified as a total 1.38 µg using Quantus Fluorometer (Promega).

### Library preparation and sequencing

Strains K0620 and K0969 were sequenced on a PacBio Revio to produce PacBio HiFi reads, intended for nuclear genome assembly, and IsoSeq reads were used to map transcriptomic data to the assembled genome. SMRTbell libraries were prepared from the HMW DNA preps of each strain using the HiFi SMRTbell Prep Kit 3.0 (PacBio Cat# 102-182-700) according to the manufacturer’s instructions, including the recommended DNA shearing step for eukaryotes. IsoSeq Kinnex libraries were prepared from total RNA using the Kinnex Full-Length RNA Kit (PacBio Cat# 103-238-700) with the IsoSeq Express 2.0 Kit for cDNA synthesis (PacBio Cat# 103-071-500), according to the manufacturer’s instructions, which include a polyA selection. The K0620 and K0969 SMRTbell libraries were barcoded and multiplexed on a single 25M SMRT cell (Cat# 102-202-200), and the K0620 and K0969 Kinnex libraries were barcoded and multiplexed on a single SMRT cell along with several other libraries. Both runs used the v.13.0.0.205983 Revio chemistry bundle and a 30 h movie time.

For strain PNB19-01, cDNA libraries were sequenced by Novogene using NovaSeq X Plus (Illumina) (3 Gb, PE150, 20M PE reads).

### Genome and transcriptome assembly, size estimation, and annotation

The PacBio genomic HiFi reads from the *N. navis-varingica* K0620 and K0969 samples were assembled into partially phased contigs using HiFiasm v.0.19.8. The assembled contigs were then screened and filtered for contamination utilizing v.0.5.0 of NCBI’s FCS-GX workflow. This pipeline integrates several tools for detecting and filtering foreign sequences. The core of FCS-GX uses Kraken 2 for taxonomic classification, rapidly aligning sequences against a curated reference database of known contaminants, including bacterial, viral, and synthetic vector sequences. In parallel, VecScreen_plus_taxonomy identifies vector and adaptor sequences based on NCBI’s UniVec database, flagging any residual synthetic contamination. The pipeline also employs BLAST-based filtering for high-specificity alignments to known foreign taxa. Contigs or scaffolds flagged by any of these tools were reviewed and removed or masked as appropriate. This approach allowed us to confidently eliminate non-*N*. *navis-varingica* DNA sequences and ensure a clean, high-quality reference genome suitable for downstream analysis and annotation.

Additionally, BlobToolKit v.4.4.4 (96) was run to identify any missed contamination within the assemblies. The sanger-tol/readmapping (97; https://doi.org/10.5281/zenodo.6563577) and the sanger-tol/blobtoolkit (98; https://doi.org/10.5281/zenodo.7949058) pipelines were used with the UniProt Reference_Proteomes_2025_02 (99) and NCBI v.5 nt (100) databases. Using the pipeline, BUSCO v.5.7.1 (101), Diamond BLASTp v.2.1.8 (102), BLASTx, and BLASTn v.2.14.1 (103) were run to taxonomically classify contigs. The filtered assemblies were then assessed for contiguity and completeness with assembly-stats v.1.0.1 and the stramenopiles_odb10 BUSCO v.5.4.3 database. Concurrently, a k-mer approach was taken to predict genome size, heterozygosity, and repeat content of the *N. navis-varingica* samples using the HiFi reads, GenomeScope 2.0, and Meryl v.1.3. (Fig. S2). For sample K0969, lower HiFi read coverage required setting the initial kmercov estimate to 10 for GenomeScope 2.0.

Transcripts were identified from the PacBio IsoSeq reads of *N. navis-varingica* K0620 and K0969 using IsoSeq v.4.2.0. The reads were segmented with Skera v.1.3.0 before primer removal and read demultiplexing via Lima v.2.12.0. After segmentation and demultiplexing, poly(A) tails and concatemers were removed with the “isoseq refine --require-polya” command. The three IsoSeq runs were then clustered for each sample by running “isoseq cluster2 --singletons” and subsequently mapped to the associated genome assembly with pbmm2 v.1.16.0.

Genome annotation was conducted using a combination of BRAKER v.2.1.6, GALBA v.1.0.1, and TSEBRA v.1.1.2.5. BRAKER was used to predict genes based on the IsoSeq transcript mapping and protein annotation sequences from eight related Bacillariophyceae species: *Phaeodactylum tricornutum* CCAP 1055/1*, Fragilariopsis cylindrus* CCMP1102*, Fistulifera solaris, Nitzschia inconspicua, Mayamaea pseudoterrestris, Pseudo-nitzschia multistriata, Seminavis robusta,* and *Cylindrotheca closterium,* with the GenBank accessions: GCA_000150955.2, GCA_001750085.1, GCA_002217885.1, GCA_019154785.2, GCA_027923505.1, GCA_900660405.1, GCA_903772945.1, and GCA_933822405.4, respectively. These protein sequences were additionally used in the GALBA annotation workflow to predict protein-coding gene structures. After BRAKER and GALBA, the results were input into TSEBRA to select the highest-confidence transcripts. Functional annotations of gene models were generated by annotation against common protein domain databases: Pfam v.35.0, PANTHER v.15.0, TIGRFAM v.15.0, KEGG v.30-01-2023, and EggNOG v.5 using a combination of tools (Diamond v.2.0.15, eggNOG-mapper v.2.1.10, InterProScan v.5.57-90.0, Kofamscan v.1.3.0) (102, 104–108). Lineage probability index was calculated from top 100 Diamond BLASTp hits by dividing the sum of probabilities of each taxonomic term by the normalization factor corresponding to its taxonomic level in the lineage and choosing the term with the highest index (109). For each assembly, RepeatModeler v.2.0.6 with default parameters was used to prepare a custom repeat library, which was used as input for RepeatMasker v.4.1.8 with the “-xsmall -nolow -norna -no_is -q” parameters.

For PNB19-01, *de novo* assembled data were used for BLAST search and other bioinformatics analyses using GENEYX-MAC (Nihon Server, Tokyo).

### Annotation of domoic acid biosynthesis gene clusters, phylogenetic analysis, motif enrichment analysis, and structural modeling

*Dab* gene amino acid sequences from *P. multiseries*, *P. multistriata*, *P. australis*, and *C. armata* were used to build a hidden Markov hodel (HMMER v.3.3.2) query for each individual gene in the cluster using hmmsearch (part of HMMER) or BLASTp (103, 110, 111). The peptide sequences from assemblies were queried to identify candidates and verified by sequence alignment.

Single-gene phylogenies were built using Kalign (EMBL-EBI), top BLAST hits for the *N. navis-varingica* sequences, representative UniRef50 sequences, in addition to those listed in previously published phylogenies (9, 112). Concatenated phylogenies of Bacillariaceae marker genes (Fig. 1) and kainoid biosynthesis genes (Fig. 4) were built using SPLACE to align and concatenate genes of interest (113). Maximum-likelihood trees were built using IQ-TREE and visualized in iTOL (90, 91). Images of *C. armata* and *D. simplex* in Fig. 1 are reproduced from our previous publication (9) with permission. These images are shown to highlight the continuity and context of the analysis of *N. navis-varingica* as a DA producer in this manuscript. The image of *C. armata* was provided by G. Saunders (University of New Brunswick), and *D. simplex* was provided by T. Teruya (University of Ryukyus).

Visualization of genomic data was performed using RStudio (v.2024.12.1.563), including the packages taxize, gggenes, and ggplot2 (114–117). Clinker was used for kainoid gene cluster comparison (118). Subsequent figure refinement (color adjustments and figure compilation) was performed in Affinity Designer (https://affinity.serif.com/en-us/designer/).

Putative *dab* integration sites from the genome assemblies of *N. navis-varingica* K0620, *P. multiseries* CNS00149, *Pseudo-nitzschia pungens* CNS00055, and *P. delicatissima* CNS00130 were aligned in nucleotide space, and the resulting consensus sequence was used for motif analysis. These sequences were selected because of the genome assembly availability and previous identification of putative *dab* integration sites (26). Motif enrichment analysis of the putative *dab* integration site was conducted using the Multiple Em for Motif Elicitation (MEME) Suite v.5.5.8 (119). Motifs were then queried against the 2024 JASPAR (non-redundant) DNA Diatom motif database (120) using the MEME Suite TomTom tool (119). Visualization of motifs was generated by the MEME Suite TomTom tool (119).

AlphaFold2 server (https://alphafoldserver.com/) was used to generate structural models of DabC and DabD proteins (60). Models were then analyzed and visualized in ChimeraX v.1.1 (62, 121). Pairwise matchmaker analysis was conducted using the default parameters (62). AlphaFold2 models were then used to model the binding pocket surface area and volume using CASTpFOLD with the default parameters (61).

### Heterologous protein expression and purification

For strain PNB19-01, *dabA* and *dabC* clones were obtained using reverse transcriptase polymerase chain reaction (RT-PCR). They were then cloned into a pET28 vector and expressed as described below. See all expressed genes in Table S4. In the RT-PCR-obtained clone of *dabA*, a single nucleotide change from 296-A (RNA-seq) to G was detected. This 296-G clone was expressed.

A putative chloroplast signal peptide was identified on K0620-dabA using HECTAR (v.1.3) and SignalP (Eukarya, v.6.0) (55, 56). Therefore, K0620 dabA was ordered with a 26-amino-acid truncation and expressed as such. Both *dabA* and *dabC* genes were codon-optimized for *Escherichia coli* expression, domesticated for SapI and BsaI cut sites, and designed with an N-terminal His-6 affinity tag in pET-28a(+) vectors and ordered from Twist Bioscience. See all expressed nucleotide sequences in Table S4.

Both DabA and DabC were expressed and purified as previously described (9, 24) using conventional methods. Constructs were transformed into chemically competent *E. coli* BL21(DE3) cells and plated on kanamycin (50 µg/mL) plates. Overnight cultures of transformed BL21(DE3) *E. coli* were used to inoculate expression cultures, which were grown at 37°C in 500 mL TB broth supplemented with 4% glycerol to an OD_600_ of ~0.6. Cultures were chilled on ice and induced with 1 mM of isopropyl β-D-1-thiogalactopyranoside. Flasks were shaken at 18°C overnight (~18 h). Cells were harvested by centrifugation (8,000 × *g*, 15 min) and frozen at −80°C until future purification.

Frozen pellets were defrosted on ice and at 4°C overnight, then resuspended in 5 mL lysis buffer (10 mM HEPES, 100 mM NaCl, 25 mM imidazole, 0.2 mM DTT, 2.5 mM EDTA, 20% glycerol, pH 7.5) per 5 mg pellet, amended with 1 mg/mL lysozyme. Cells were lysed by sonication using a Qsonica tip at 50% amplitude for 15 s on, 45 s off, 7 min total working time. DNase I in 5 mM MgCl_2_ was added halfway through sonication to a final concentration of 5 µg/mL. Lysate was centrifuged at 40,000 × *g* for 30 min at 4°C to remove cellular debris. The supernatant was filtered through a Whatman filter before purification.

Purification was performed using immobilized metal-affinity chromatography purification of His6-tagged proteins using a HisTrap FF column (Cytiva) on an AKTA pure 25 L1 (Cytiva) fast protein liquid chromatography (FPLC) system and a BioLogic DuoFlow system (Bio-Rad). FPLC data were analyzed with UNICORN version 7 software. Clarified lysate was loaded at 2 mL/min onto a 5 mL HisTrap FF column (Cytiva) pre-equilibrated with lysis buffer. The column was washed with 10 column volumes of 8% elution buffer (10 mM HEPES, 100 mM NaCl, 500 mM imidazole, 20% glycerol, pH 7.5) and then eluted with a linear 8%–100% gradient over 15 column volumes in 4 mL fractions. Fractions were analyzed by SDS-PAGE. Fractions containing the target protein were pooled, desalted, and buffer-exchanged using PD-10 columns (Sephadex G-25 M, Cytiva) that were pre-equilibrated with storage buffer (50 mM HEPES, 250 mM NaCl, pH 8, 10% glycerol). Storage buffer for DabA was amended to a final concentration of 5 mM MgCl_2_.

Buffer-exchanged protein was concentrated using Amicon Ultra-15 centrifugal filters, aliquoted, and flash-frozen in liquid nitrogen. Aliquots were stored at −80°C until further analysis. All protein quantification was calculated using denatured protein UV absorbance at 280 nm and the protein’s extinction coefficient, at multiple dilutions with Milli-Q water.

### Preparation of substrates and standards and enzymatic activity assays

DA standard was purchased from the National Research Council of Canada (122). Kainic acid standard was purchased from Chem-Impex. Preparation of all non-commercial substrates was used as prepared in previous studies (9, 25, 30, 33). All substrates and enzymatic assay products were verified using retention time and mass via LC-MS.

DabA enzyme assays to demonstrate *N*-prenyltransferase function were performed as previously described (9, 24, 31), with a few modifications. The reaction mixture was prepared in a final volume of 100 µL in 100 mM HEPES (pH 8.0), 100 mM KCl, 10% glycerol buffer with 5 mM MgCl_2_, 1 mM geranyl diphosphate, and 20 mM of L-glutamate. Reactions were allowed to incubate at room temperature (~22°C) for 6 h and were then quenched with 100 L of ice-cold methanol. Quenched reactions were centrifuged, filtered, and injected (10 µL) onto LC-HRMS.

Enzyme assays to demonstrate kainoid synthase activity for K0620-DabC and PNB19-01-DabC were performed as previously described (9, 24), with few modifications. A reaction mixture was prepared in a final volume of 100 µL containing 100 mM HEPES (pH 8.0), 100 mM KCl, 10% glycerol, 1 mM L-ascorbate, 6.25 mM 2-oxoglutaric acid, 25 µM to 1 mM of either 7′-COOH-L-NGG or L-NGG, 50 µM DabC, and 50 µM FeSO_4_·7H_2_O, and incubated at 25°C overnight (15 h). The reaction was quenched by adding 100 µL of methanol, followed by centrifugation. The supernatant was purified via filtration with a nylon 0.22 µm pore CA membrane (Costar Spin-X) or purified using a reversed-phase resin (Cosmosil 140C-OPN) prior to LC-MS analysis.

### Metabolite extraction from *N. navis-varingica*

Solid-phase extraction was performed using Agilent Bond Elut PPL cartridges (200 mg, 3 mL), in a manner similar to analysis for dissolved organic matter (123). During the exponential phase of strains K0620 and K0969, 50 mL of cell culture was acidified to pH 2 with concentrated HCl. The PPL cartridges were washed and activated using LC-MS grade methanol and LC-MS grade water (pH 2), respectively. To capture the whole water (both particulate and dissolved fractions) metabolome, acidified samples were loaded onto the cartridge under vacuum in a drip-wise manner. After sample loading, cartridges were washed with acidified LC-MS grade water (pH 2) to remove salts. Cartridges were dried under nitrogen gas and eluted with 2 mL of LC-MS grade methanol. Samples were dried down in a Speedvac and resuspended in 100 µL of 80% aqueous methanol (LC-MS grade) with 0.1% formic acid (LC-MS grade). Samples were stored at −80°C until LC-MS analysis.

PNB19-01 cell extract (the 1 month culture, 8 mL; approximately 10 mg cells) was collected by centrifugation at 1,500 × *g* for 3 min, then extracted with 50% MeOH 100 µL by sonication 10 s. After centrifugation (20,600 × *g,* 30 s), the supernatant was collected. Half of the supernatant (approximately 50 µL) was used after removal of the solvent by vacuum centrifugation at room temperature (finally from 5 mg cell extract).

### Liquid chromatography-mass spectrometry

LC-MS measurements were conducted in a similar manner as previously described (13). Samples were injected onto an Agilent single quadrupole UPLC-MS iQ using the Single Quadrupole Analytical LC-MS. Compounds were separated by reversed-phase chromatography on a Phenomenex Kinetex 5 µm C18 100 Å 150 × 4.6 mm LC column with water + 0.1% formic acid (solvent A) and acetonitrile + 0.1% formic acid (solvent B) as eluents. The following gradient was applied at a flow rate of 0.75 mL/min: hold at 5% B for 1 min, 5% to 35% B over 30 min, 35% to 100% B over 1 min, hold at 100% B for 1.5 min, 100% to 5% B over 2.5 min, hold at 5% B for 2 min.

Higher-resolution mass spectrometry was necessary for detection of biosynthetic intermediates in culture extracts. High-resolution LC-MS measurements were conducted using an Agilent Technologies 1200 Series system with a diode array detector coupled to an Agilent Technologies 6530 accurate-mass Q-TOF LC-MS. Identical chromatographic methods were applied and run in negative ionization mode.

## Data Availability

The raw sequencing data (accessions: SRR34800284, SRR34800285; BioProject: PRJNA1299467) and annotated *dab* gene cluster contigs (accessions: PX214389, PX214390) from *N. navis-varingica* strains K0620 and K0969 are available via NCBI. Partially phased assemblies are available on FigShare (https://doi.org/10.6084/m9.figshare.29913065.v1). Sequences from strain *N. navis-varingica* strain PNB19-01 are available via DDBJ under accession numbers LC887833–LC887838.

## SUPPLEMENTAL MATERIAL

Supplemental figures and tables mbio.02079-25-s0001.pdf Tables S1 to S4; Figures S1 to S19.

ASM does not own the copyrights to Supplemental Material that may be linked to, or accessed through, an article. The authors have granted ASM a non-exclusive, world-wide license to publish the Supplemental Material files. Please contact the corresponding author directly for reuse.
