# Supplementary material for: Domoic acid biosynthesis and genome expansion in Nitzschia navis-varingica
Source: mBio. 2025 Oct 30;16(12):e02079-25. doi: 10.1128/mbio.02079-25 (PMC12691652; doi:10.1128/mbio.02079-25)
Supplement: Supplemental figures and tables — Tables S1 to S4; Figures S1 to S19. [file mbio.02079-25-s0001.pdf]

**Supporting Information**

**<u>Domoic acid biosynthesis and genome expansion in *Nitzschia navis-varingica*</u>**

Steffaney M. Wood-Rocca,[1,2] Nicholas Allsing,[3] Yasuhiro Ashida,[4] Masaki Mochizuki,[4] Malia L. Moore,[1,3] Zoltán Füssy,[2,5] Yuichi Kotaki,[4] Clyde Puilingi,[6,7] Yukari Maeno,[8] Aodhan W. Beattie,[1] Andrew E. Allen,[2,5] Mari Yotsu-Yamashita,[4] Todd P. Michael,[1,3] Bradley S. Moore[1,9]*

1 Center for Marine Biotechnology and Biomedicine, Scripps Institution of Oceanography, University of California San Diego, La Jolla, California, USA

2 Environmental Genomics group, J. Craig Venter Institute, La Jolla, California, USA

3 The Plant Molecular and Cellular Biology Laboratory, Salk Institute for Biological Sciences, La Jolla, California, USA

4 Graduate School of Agricultural Science, Tohoku University, Aramaki-Aza-Aoba, Aoba-ku, Sendai, Japan

5 Integrative Oceanography, Scripps Institution of Oceanography, University of California San Diego, La Jolla, California, USA

6 Faculty of Science and Technology, Solomon Islands National University, Honiara, Solomon Islands

7 School of Science & Technology, Pacific Adventist University, Port Moresby, Papua New Guinea

8 Graduate School of Agricultural and Life Sciences, The University of Tokyo, Bunkyo-ku, Tokyo, Japan

9 Skaggs School of Pharmacy and Pharmaceutical Sciences, University of California San Diego, La Jolla, California, USA

*Corresponding author emails: bsmoore@ucsd.edu, tmichael@salk.edu

**Table of Contents**

**Supplementary Tables**


| | |
|---|---|
| Table S1. Genome statistics for *N. navis-varingica* assemblies | **3** |
| Table S2. Genome statistics before and after contamination removal | **4** |
| Table S3. Iodoperoxidase sequence and BlastP results | **5** |
| Table S4. Nucleotide sequences for genes expressed in this study | **6** |


**Supplementary Figures**


| | |
|---|---|
| Figure S1. Bacillariaceae family phylogeny | **7** |
| Figure S2. Genome survey analysis | **8** |
| Figure S3. Contamination removal from assemblies | **9** |
| Figure S4. Distribution of functional annotations | **10** |
| Figure S5. Iodotyrosine deodinase contig | **11** |
| Figure S6. Domoic acid gene cluster contigs | **12** |
| Figure S7. *dabC2* contig | **13** |
| Figure S8. DabD sequence comparison | **14** |
| Figure S9. Segment repeat analysis | **15** |
| Figure S10. Phylogenies of protein kinase and CoA-binding genes | **16** |
| Figure S11. Putative integration site motifs | **17** |
| Figure S12. Domoic acid isomers in culture extracts and NnvDabC assays | **18** |
| Figure S13. NnvDabA assays | **19** |
| Figure S14. DabC L-NGG assays | **20** |
| Figure S15. Dainic acid in culture extracts | **21** |
| Figure S16. DabC and DabD phylogenies | **22** |
| Figure S17. DabB phylogeny | **23** |
| Figure S18. *N. navis-varingica* CYP450 phylogeny | **24** |
| Figure S19. AlphaFold2 and CastpFOLD analysis | **25** |

**Table S1.** Genome statistics for assemblies produced for *N. navis-varingica* K0620 and K0969. Percentage repeats and coding sequences not calculated for consensus v1 assemblies.

| Assembly | K0620 consensus (v1) | K0620 hap1 | K0620 hap2 | K0969 consensus (v1) | K0969 hap1 | K0969 hap2 |
|---|---|---|---|---|---|---|
| Size (Mbp) | 1140.75 | 862.3 | 719.1 | 1047.0 | 766.5 | 624.8 |
| Number of contigs | 4,911 | 5,895 | 3,355 | 8,655 | 9,152 | 6,055 |
| N50 (Mbp) | 0.443 | 0.304 | 0.343 | 0.198 | 0.136 | 152,664 |
| BUSCO Complete (%) | 99 | 94 | 92 | 96 | 85 | 87 |
| BUSCO Duplication (%) | 43 | 19 | 20 | 55 | 30 | 17 |
| GC content (%) | 31.75 | 31.95 | 31.24 | 33.37 | 33.89 | 33.77 |
| Repeat region (%) | - | 71 | 70 | - | 70 | 71 |
| Coding sequences (%) | - | 7.17 | 7.15 | - | 7.12 | 7.21 |

**Table S2.** The genome statistics before and after filter contamination.

| | | Initial assembly | FCS-GX | Blob-tools |
|---|---|---|---|---|
| K0620 | Genome size (Mbp) | 945.8 | 879.5 | 862.3 |
| | Filtered (Mbp) | | 66.3 | 17.2 |
| | N50 (Mbp) | 0.305 | 0.304 | 0.306 |
| K0969 | Genome size (Mbp) | 904.2 | 782.8 | 766.5 |
| | Filtered (Mbp) | | 121.4 | 16.3 |
| | N50 (Mbp) | 0.151 | 0.134 | 0.136 |

**Table S3.** Amino acid sequence for putative iodoperoxidase gene from K0620 hap1 assembly. Top BlastP hits from NR database.

<table>
<tr><td colspan="5">>NnvK0620hap1-putative-iodoperoxidase<br>MFCKKYDDESNNVVEFQSGQVEPKHGGNDNNNNTATTTFHVPYHHQRLSIQDIRIRAQKFLRLLSMRRTI<br>RFFSSDPIPKDIIETIIQTAATAPSGAHKQPWSFVAVSNPEIKAKIRIMVEEEETINYQRRMRKSWVKDVEGM<br>VSQVHASSGGTNSGGGGGGGMTETATATSGDGSGSGSSSSPNDTKKDVVIQKPYLTEAPWLIVVFKQPHG<br>GVDPITNERIDHYYVQESVGIACGMLISAIQNANLATLTSTPMGAEKGIRNLLGRPEHEKVVLLMPVGFPA<br>KHATVPYRHPERKPLDQVLSWKC</td></tr>
<tr><td>Result description</td><td>Scientific name</td><td>E-value</td><td>Percent ID</td><td>Accession</td></tr>
<tr><td>Iodotyrosine deiodinase 1</td><td>Seminavis robusta</td><td>2e-115</td><td>57.67</td><td>CAB9517703.1</td></tr>
<tr><td>Iodotyrosine dehalogenase 1 precursor, putative</td><td>Perkinsus marinus ATCC 50983</td><td>2e-69</td><td>48.22</td><td>XP_002768445.1</td></tr>
<tr><td>hypothetical protein FOZ61_010198</td><td>Perkinsus olseni</td><td>2e-68</td><td>45.74</td><td>KAF4666096.1</td></tr>
</table>

**Table S4.** Nucleotide sequences of genes expressed *in vitro*.

| | |
|---|---|
| dabA_K0620_DN15972_NO_SIGNAL P | ATGGTTAACGGCGATGGCTACGGTTCAGTTAATGGATCTGTAAACGGTTCTGTAAACGGCAAGGTCAATGGATCTATCAATGGGGTGAATGGAGTGAACGGTGTTAACGGTAAGCTGAACGGTGTGAATGGTAACATC<br>AATATTGTCAACGGTAACGCGATCGGTTTCAGCTCACCATCAGAAAAGCCAGAGGAAGCCTTAGCACGATTTCAAAAGAATGGGCTGACCCTCACTAACCCGAACGATCTGTATTGGATGAACGCTTTCCTCAAGGAG<br>AACTTCTACAATAAGGGGAATTACTATTACCCAATTAAGACCATTTGTGACGGCGAGCTGATTGACGTAACTTTCTACTGCCCATTTGAGCCAAATATTAGCCCTCACTATACTAAGCTTTACAATACACGCGACGAAC<br>GTAGTACTCTGTTTGCAATTCCCCCAGCGAAAACAGAAATGACAAAGTACAACCGTATCAACTGTGAGAAAACCTCTGGATTAACGGCACCATTTTCTAGCTTTGAGGATACCCAAATGGTAGCGTTCTTCTATAGCAT<br>GATGTACTACCTGAATGATCAGACTGCCCATCTGAAGTTGCCAGAGGAAGAGATACAGCCAGAGCTCGTCGATGAGTTGAACGATCATTTACTTCTTTACCTGGGCGAGTTCCTGGCTCTTATGAAATACGCAGATTCA<br>GAGGACATTGACCGAATTTGGGACTACTTAGACTTCTACCAGCCGTATTTCCACAAGGTGGACGGGGAAGGGATTTTCTTGGACAAGAAGTACCAAGGTCAAACGCCCCCGCAAATACGCTTGATCCAGACAATGAAC<br>AACTACATCGTCGAAAACTTCGCGTCAACGAAGAATATCTCCCAAGTAGTATACGAAGTTGTTAAGTATATCAAGGGTATCAAGGATGAAATTAAAATCCGCGGGGATAAGAGTTTCACCCTGTCCCTGCAAGAGTAC<br>GACGAATTTCGGGACAAGGTGACCGCCAGCCCGATGGCTCACGCTATCACTGATCTGACACACCCCTACATTAGCTACAAAGCGTATCAAGACCCGCTTTTCATAAGACTGGAGAACCTGGCTTGCGAGATTATCACTT<br>ACTTTAACGATGTGTGCACGTCTGATCGCGAACGCCTTGACGACGACCCATTCAACTCAGTATTTATCTTACGTGACCTTGACAGTAGCCTGTCGTATGCGCAAAGTTGTGATATAGTTGTCAAGATTGCTCACGAAAAG<br>ATGGAGAAATTCTTAGATGTTAAGAAGCAATTAATAGAAGCCGCATCGCACGATGAATACGAATTGAAGGCAATAACTCAAATGATCAAAACGAGAGAGGATTCGCTGATTGGTTACGTTTGCCACGAGGTGTGCTGT<br>GTCGAGAACGGGTACGCGCGAGATCATAAGCCGCTCATGAAGGAGTACTTAGAGGGGCAGATAGTCGAACATCTTCAGAAACAACTGCAACTCAGTTAA |
| DabC_K0620_IsoSeq_248443.p1 | ATGAACATAGATCACATGAAGTTCAGCCCGCTGGAAGCCGAGAAGTTAAAGTGGTACCCGAAGTCCTCACTCCCACCAGAAATACCGGCCATAGATATATCCAAAGTGAACACTGACGAGGAGCTTATTCAGTTCCTG<br>GCTGACATCAGAAAGAGTGGTCTGTTTTACATTGTAGGGCACGGCATACCCGAGGAAGTTCCGATGAACACCTATAATTCGTTCAGAGAATTCTGCAACCTGCCTGAAGAAACCAAGATGACTTATCACACCGATCCAT<br>TCTTCCGCAATGGTGGATACGTCCCGTTCGCTGGCAGCAGTATCGCCGGCGGGAATCAGGACAAGAAGGTACGAGACTGGGTTACGAAATACTTCTGGCGTGGCCCTTCGGTTGTAAACCGCTGCCCGAACGACAAGT<br>TTCAAGAAATCCACGATGAATACCACCGCCGAACACACGACCTTGCGGAGAGAATTATACGCACGATATTACGCGCCCTGAAGGCTCGTTTCTCGGACTTTGATCCTGCCGAGTACGAAGAATTTATCAATGCCGAGA<br>ACATGTTCTTCTCTAATAGAATTTACCCTGAAATTCCGCCGGACAGCGAAGAAACCGCTCAACACCGCCTTGTTCCTCACCGTGATTTATCCTGGATCACTCTGGCAAACCAGACCCCTGCTGACAACGGCTTTAAGGG<br>GTTATTTGTGGTCAAGAATGATGGCTCGAAAGTACATGTGCCCCCAATTCGCAACAGCTACTTAGTGTTCATCGGTCAATCCATGTCCTATATGACGAAGAAGTTTATTCCAGCTGTGTACCATGGCGTTGACGGGCCCC<br>CGCTGGATCAGTTGGAAGGATCAGAGAGAAGCGCACTGATTTGTTTCTACGAGCCTCACAACATAATGAACCCTTCGCGTAATATAAACCCTAATGCCGACGAAATCGACGAGAAAGGCTGCACTTTCTACGACGCAT<br>GTGGCGCCGATGAAAGTGGTACCACGTGGAAGTACGTGTCATCCAAGTTCATTGACGGTTACTTTTGA |
| PNB1901NitdabA | ATGATTCTTG GGTTGAAAAA TGCGAAGATG GTCTGGTCGG CATTTGCCGC AACCTCGACC ACAACTTCGC TTAATGCCAT GTATAAGGAT GGGGAAATCA ATGGTAACGT CAATGGAAAA GTTAATGGTG<br>TCAATGGAAT CGAGAGCATA AATGGGGTCA ATGGGAACGC CGTTGAATTA TCTGGCCCCA CAGAAACACC TGAAGAGGCA CTTGCTCGTT TTCAAAAGAA TGGGCTTACG CTGACTACTC CAAATGACTT<br>GTACTGGATG TCTGCGTTCT TGAAGGAAGA ATTTTACGAG AAGGGCGATT ATTATTACCC AATTAAAACC ATTTGTGATG GTGAGCTTAT TGACGTTAAA TTTTATTGCC CATTTGAACC AAATATCAGT<br>CCCCATTACA CCAAACTGTA CAACACTCGT GATGAAAGAT CGAGTTTGTT CGCCATCCCC CCGGCCAAAA CCGAAATGAC CAAGTACAAC CGAATTAATT GTGAAAAAAC TTCTGCTTTG ACCGCTCCTT<br>CCTCAAGTTT TGAAGACACC CAAATGGTTG CATTCTTTTA CTCTATGATG TACTACCTAA ATGACCAAAC TGCCCACCTC AAGCTCCCCG AAGAAGAAAT CCAACCTGAA CTTGTCGATG AACTCAATGA<br>TCATCTTTTG CTTTACTTGG GCGAGTTCCT CAGCCTTACG AAGTACTCAA ATAGCGAGGA TATCGAAAGA ATTTGGGATT ATTTGGAATT CTACCAACCC TACTATCAAA AGGTCGAGGG CGAGGGAATT<br>GTCTTGAAAG AGAAATACCA AAGAGAAGCT CCGCCACAAA TTCAACTAAT CCAGACGATG ACAAACTACA TCGTAGAAAA TTTCGCTTCT ACCAAGAACA TATCTCAAGT TGTTTATGAA GTTATCAAGT<br>ACATCAAGGG TATCAAAGAC GAAATCAAAA TTCGGGGCGA CAAGAGCTTC ACCCTATCGC TCAAGGAATA CGATGAGTTC CGAGACAAGG TTACAGCAAG TCCTATGGCT CATGCCATTA CTGATTTGAC<br>TCATCCGTAC GTTTCCTATA AGGCCTACCA AGACCCTCTT TTCATTCAGT TGGAAAATCT TGCTTGCGAA ATTATTACCT ACTTCAATGA TGTATGCACA AGCGACAGAG AAAGGTTGGA CGATGATCCT<br>TTCAACTCAG TCTTCATTTT GCGTGACCTT GATAGTAGTT TGAGCTACGC CCAATCATGT GATATTGTCG TCA |
| PNB1901NitdabC | ATGATTCTTGGGTTGAAAAATGCGAAGATGGTCTGGTCGGCATTTGCCGCAACCTCGACCACAACTTCGCTTAATGCCATGTATAAGGATGGGGAAATCAATGGTAACGTCAATGGAAAAGTTAATGGTGTCAATGGA<br>ATCGAGAGCATAAATGGGGTCAATGGGAACGCCGTTGAATTATCTGGCCCCACAGAAACACCTGAAGAGGCACTTGCTCGTTTTCAAAAGAATGGGCTTACGCTGACTACTCCAAATGACTTGTACTGGATGTCTGCGT<br>TCTTGAAGGAAGAATTTTACGAGAAGGGCGATTATTATTACCCAATTAAAACCATTTGTGATGGTGAGCTTATTGACGTTAAATTTTATTGCCCATTTGAACCAAATATCAGTCCCCATTACACCAAACTGTACAACACT<br>CGTGATGAAAGATCGAGTTTGTTCGCCATCCCCCCGGCCAAAACCGAAATGACCAAGTACAACCGAATTAATTGTGAAAAAACTTCTGCTTTGACCGCTCCTTCCTCAAGTTTTGAAGACACCCAAATGGTTGCATTCTT<br>TTACTCTATGATGTACTACCTAAATGACCAAACTGCCCACCTCAAGCTCCCCGAAGAAGAAATCCAACCTGAACTTGTCGATGAACTCAATGATCATCTTTTGCTTTACTTGGGCGAGTTCCTCAGCCTTACGAAGTACT<br>CAAATAGCGAGGATATCGAAAGAATTTGGGATTATTTGGAATTCTACCAACCCTACTATCAAAAGGTCGAGGGCGAGGGAATTGTCTTGAAAGAGAAATACCAAAGAGAAGCTCCGCCACAAATTCAACTAATCCAGA<br>CGATGACAAACTACATCGTAGAAAATTTCGCTTCTACCAAGAACATATCTCAAGTTGTTTATGAAGTTATCAAGTACATCAAGGGTATCAAAGACGAAATCAAAATTCGGGGCGACAAGAGCTTCACCCTATCGCTCA |

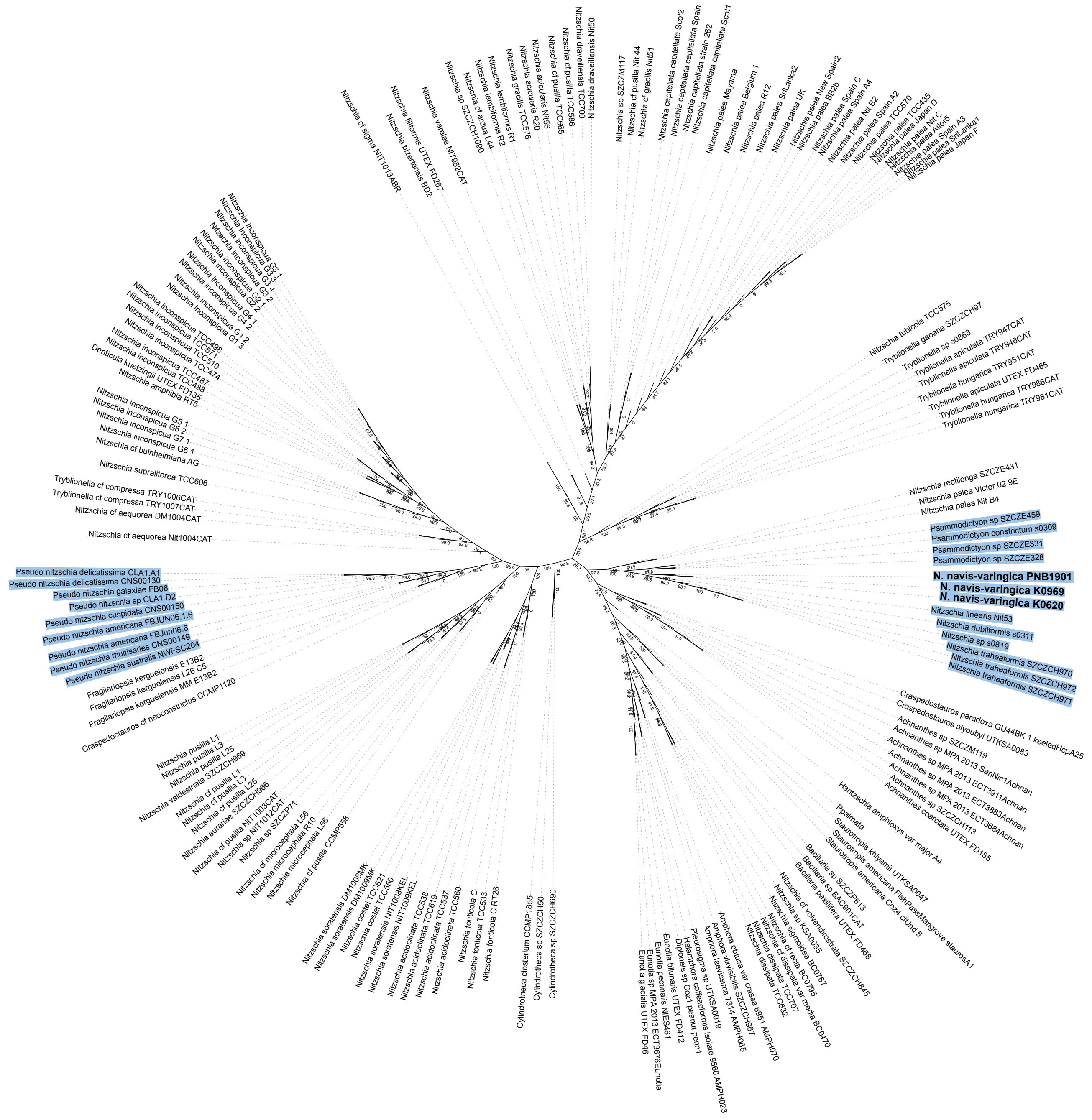


**Figure S1.** Rooted phylogenetic analysis of Bacillariaceae family using concatenated alignments of 18S (SSU) and 23S (LSU) nuclear RNA genes as well as chloroplast genes psbC and rbcL. *Digenea simplex* genes used as outgroup, which is not shown for scale. Blue clades are included in Fig. 1. Bolded are strains highlighted in this study.

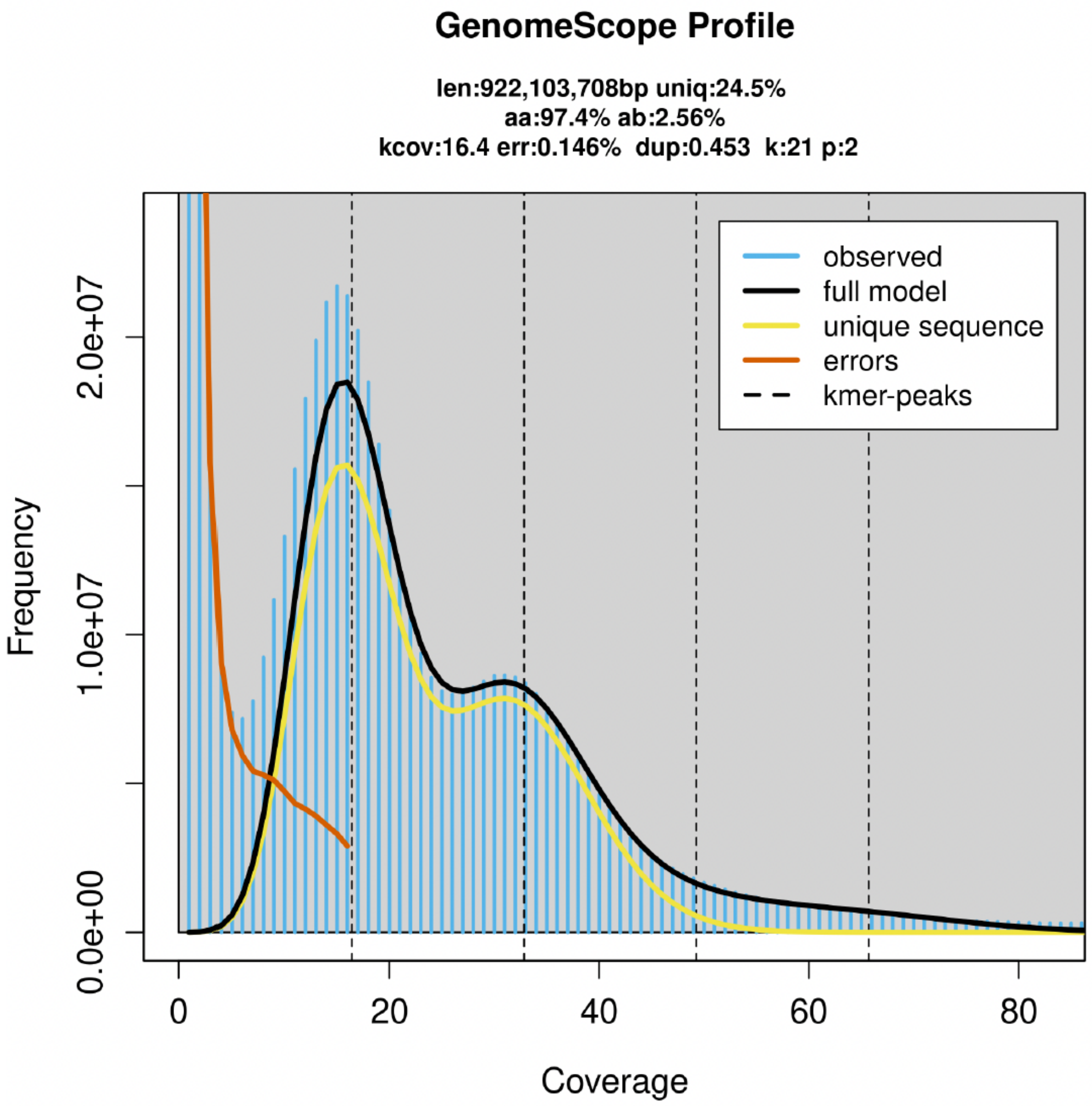


**Figure S2.** Genome survey analysis of *N. navis-varingica* K0620

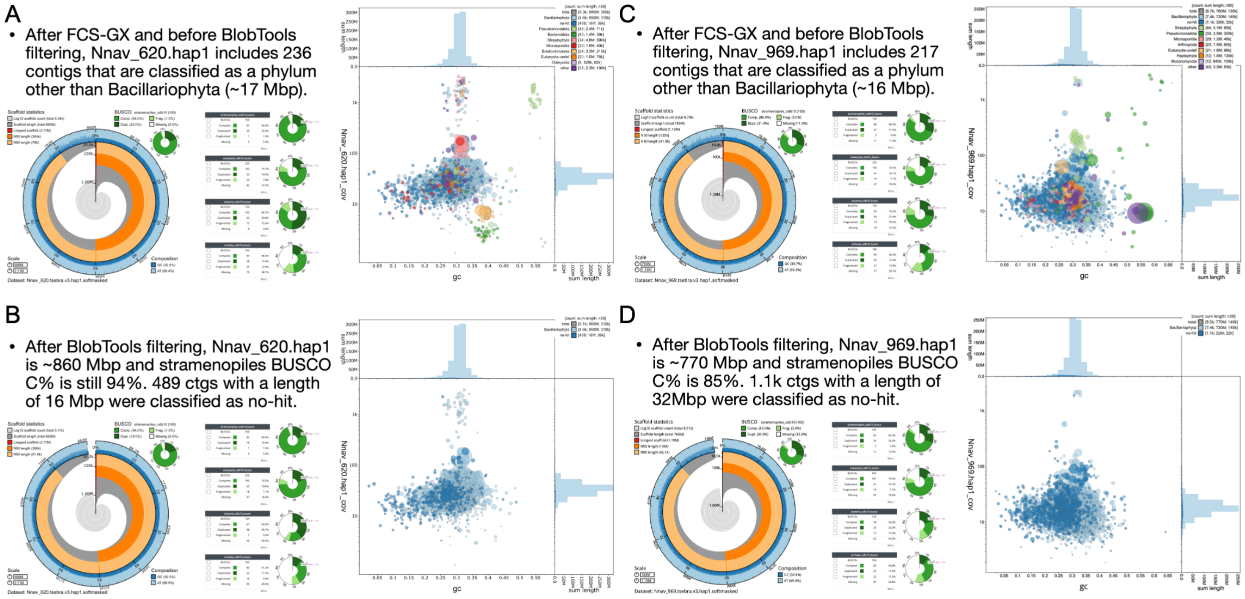


**Figure S3. Contaminant Detection and Removal Using FCS-GX and BlobTools in *N. navis-varingica* Haplotype 1 Assemblies.** BlobTools taxonomic binning and GC-coverage plots for *N. navis-varingica* haplotype 1 assemblies before and after contaminant filtering. (A, C) Assemblies of strains K0620 (A) and K0969 (C) following NCBI Foreign Contamination Screening-GX (FCS-GX) but prior to BlobTools filtering, showing the presence of 236 and 217 contigs (~17 Mbp and ~16 Mbp), respectively, classified as belonging to non-Bacillariophyta phyla. (B, D) Assemblies after BlobTools filtering, resulting in a final genome size of ~860 Mbp for K0620 and ~770 Mbp for K0969. BUSCO completeness (Stramenopiles dataset) remained high (94% for K0620; 85% for K0969), with 489 and 1,100 contigs (~16 Mbp and ~32 Mbp, respectively) classified as "no-hit," likely representing unresolved or novel sequence. Circular scaffold statistics, BUSCO summaries, and GC-content vs. coverage scatter plots are shown for each assembly.

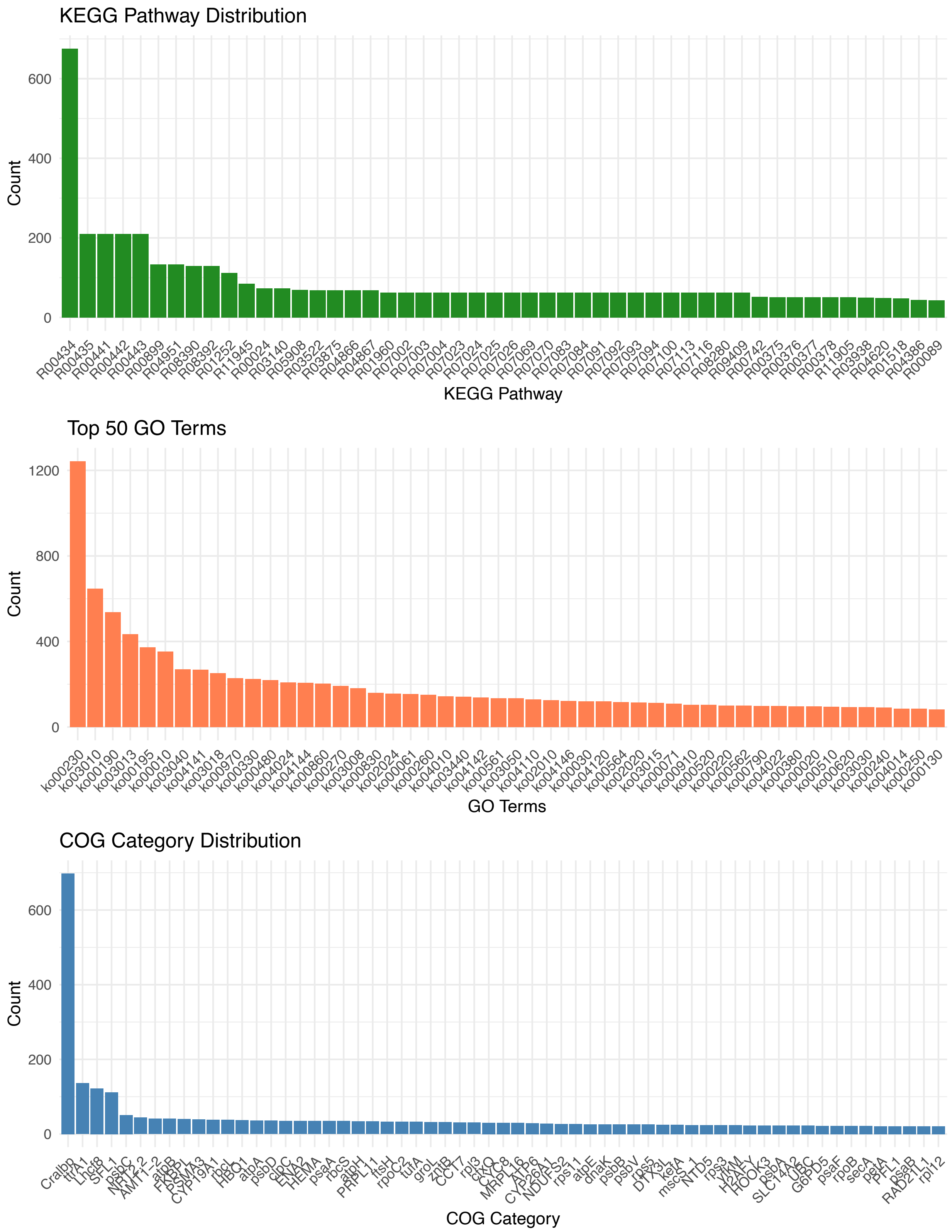


**Figure S4.** Distribution of KEGG, GO, and COG terms in EggNOG mapper functional annotations.

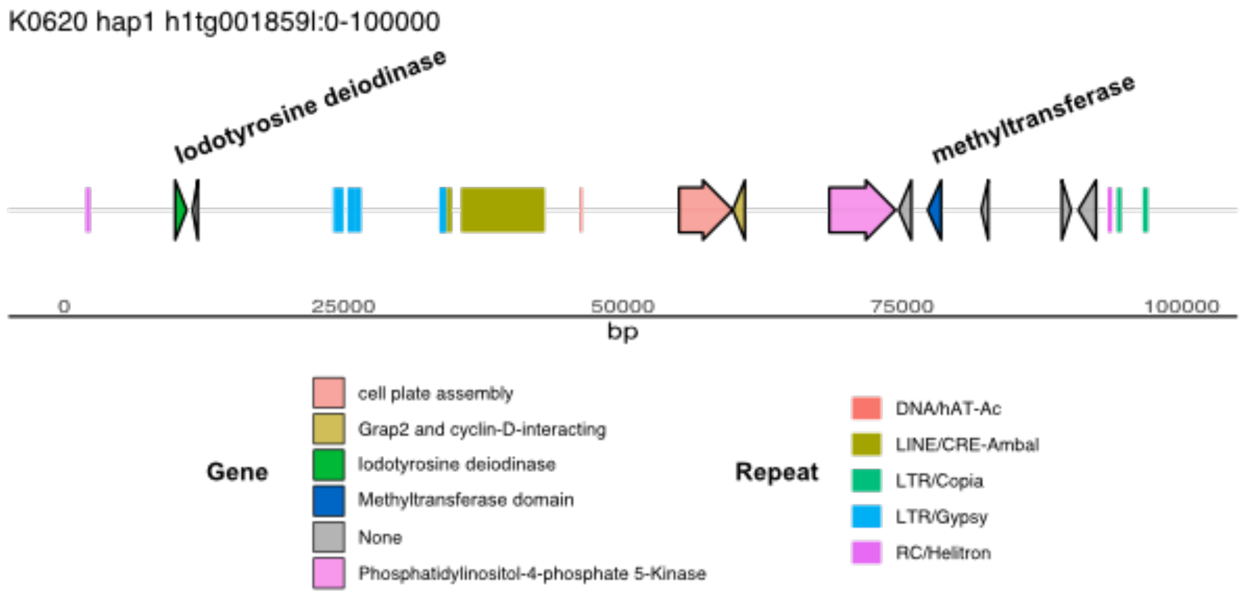


**Figure S5.** Putative iodotyrosine deiodinase gene containing contig in K0620 hap1. Arrows represent genes and bars represent repetitive elements.

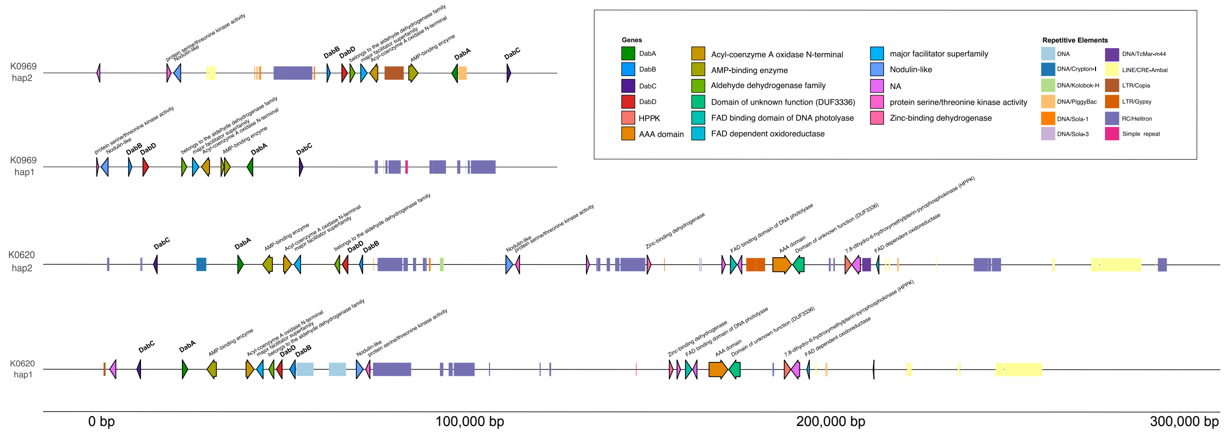


**Figure S6.** Domoic acid biosynthesis gene cluster containing contigs across both haplotype-resolved assemblies for strains K0620 and K0969. Arrows represent genes and bars represent repetitive elements.

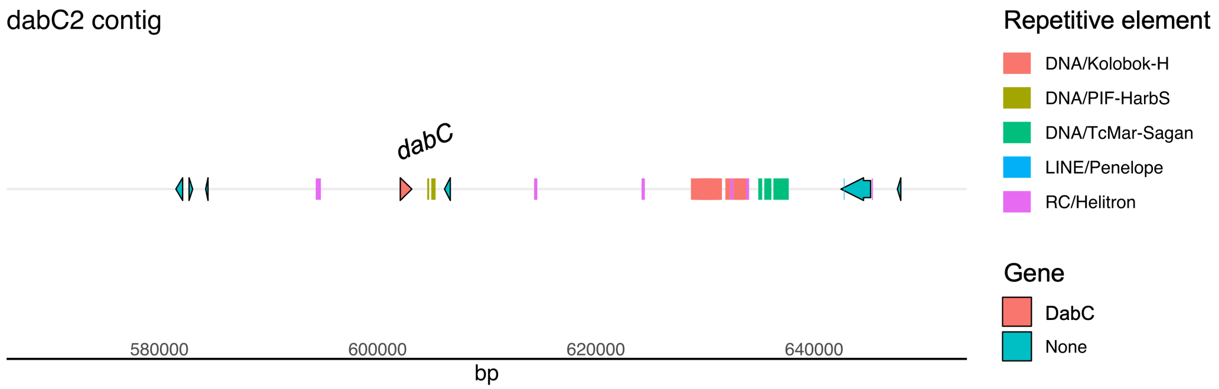


**Figure S7.** *N. navis-varingica* hap1 *dabC2* contig genomic neighborhood. Arrows represent genes and bars represent repetitive elements.

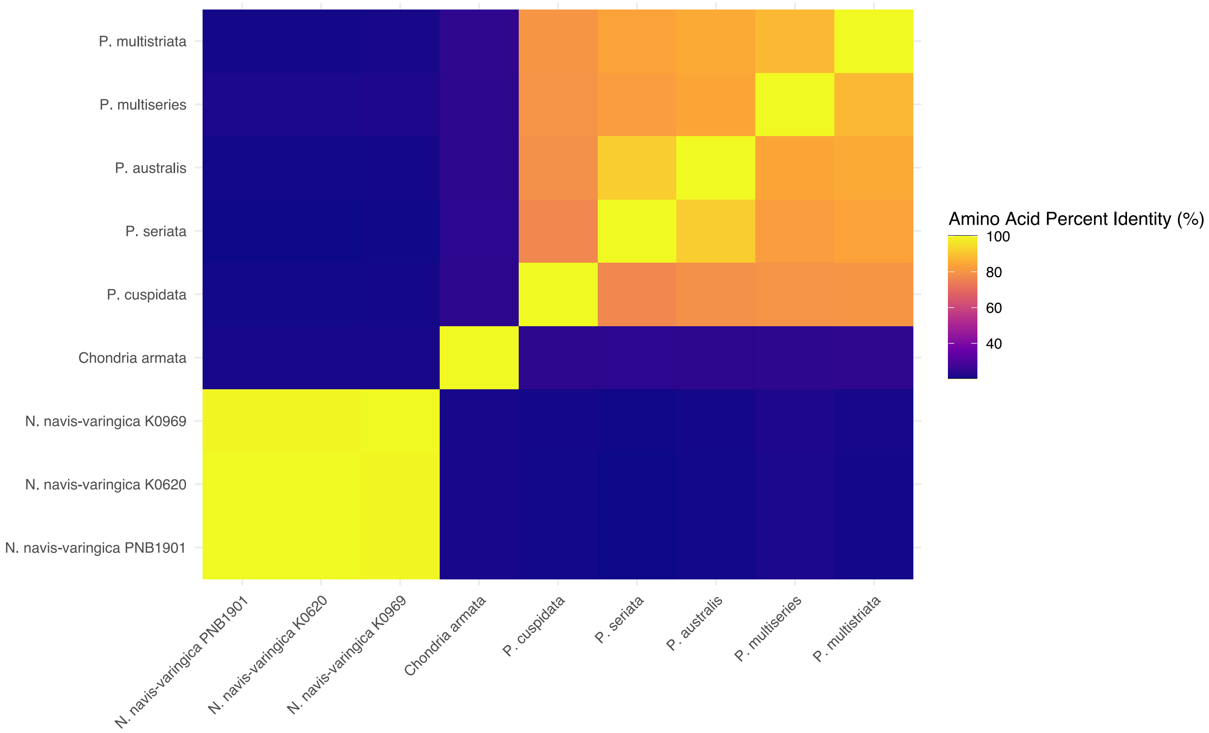


**Fig S8.** Heatmap of amino acid percent identity among DabD sequences.

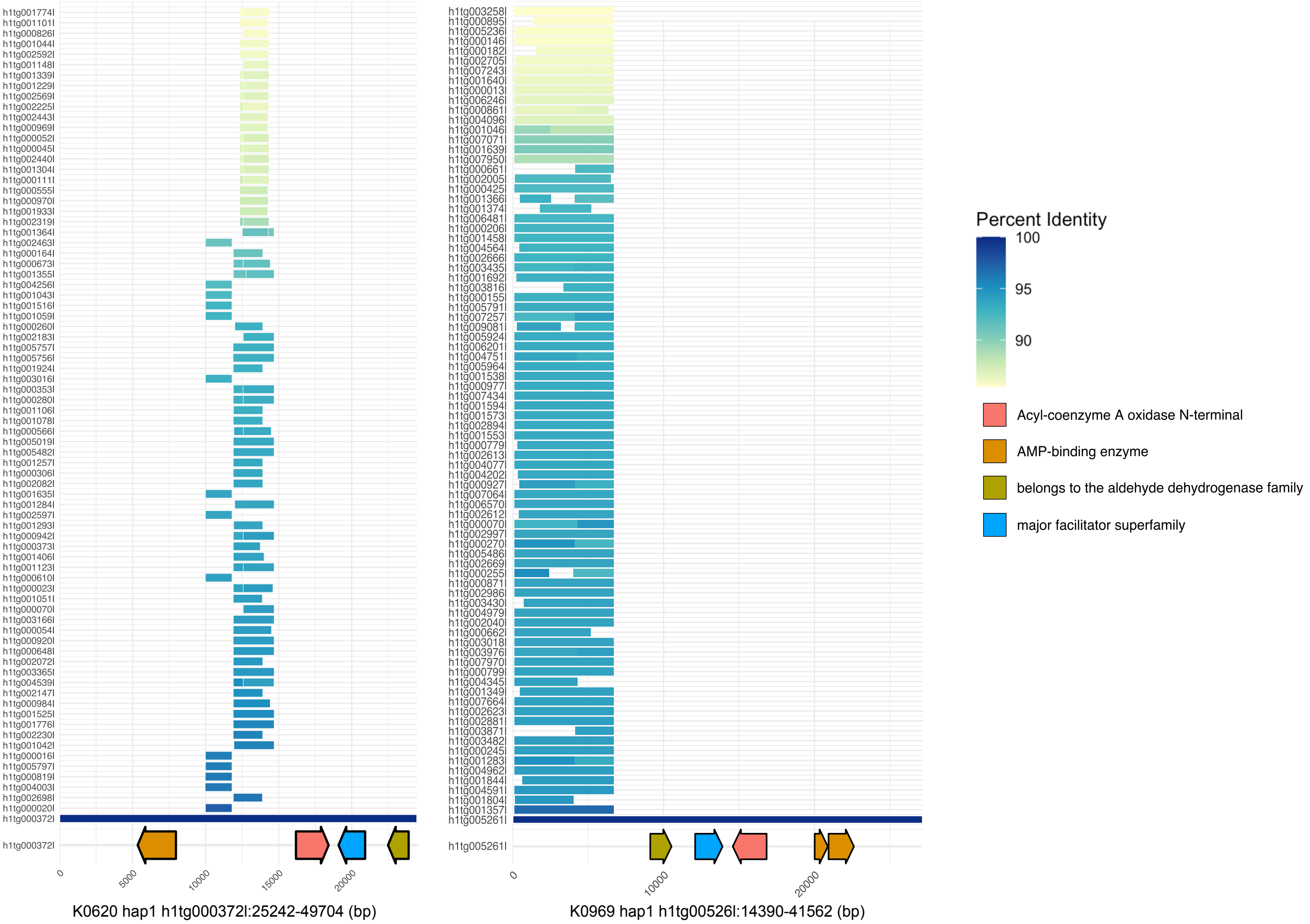


**Figure S9.** Comparative analysis of top 100 BLAST hits for *dab* cluster segment repeat element in K0620 hap1 and K0969 hap1. Each row represents a contig, horizontal bars show the aligned portion of the segment repeat and indicate nucleotide percent identity (85%+) relative to the reference *dab*-containing contigs shown on the bottom row. The schematic at the bottom illustrates the portion of the *dab* cluster between *dabA* and *dabD,* with coding sequences represented as arrows.

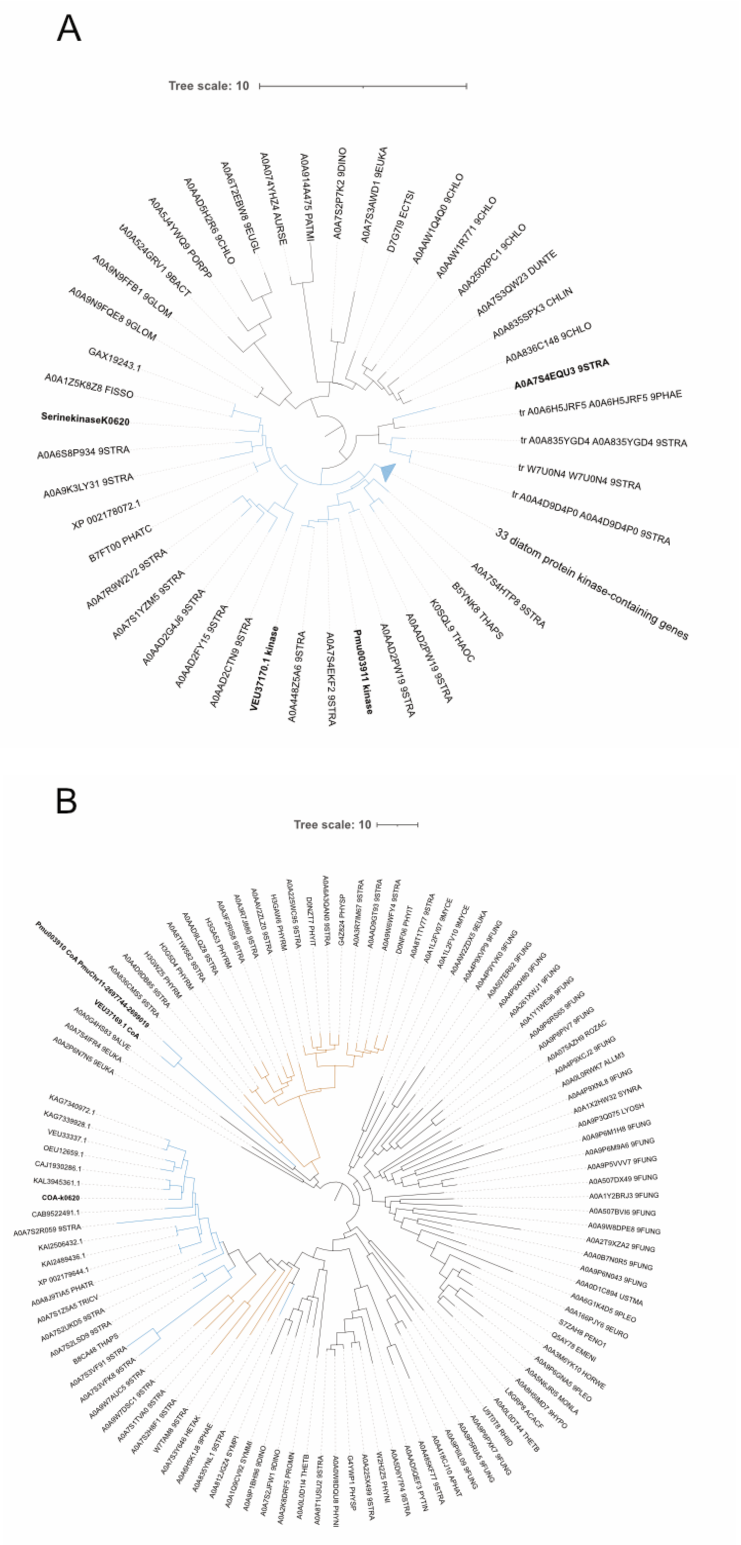


**Figure S10.** Phylogenetic analysis of (A) protein kinase and (B) CoA-binding protein genes. *Nitzschia navis-varingica* and *Pseudo-nitzschia* spp. genes are bolded. Blue branches correspond with diatom sequences; Brown branches correspond with other Stramenopile sequences.

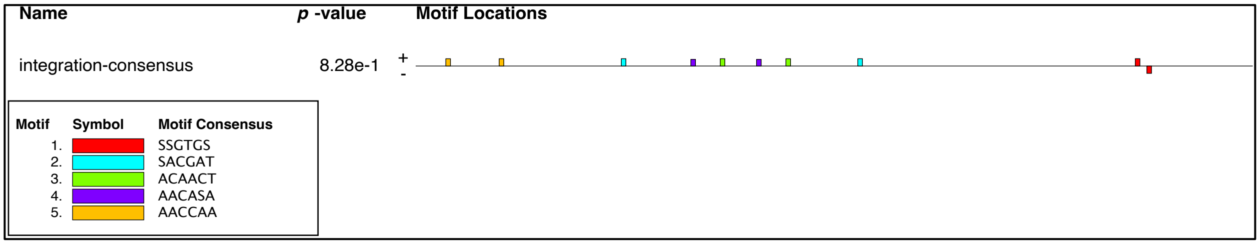


**Figure S11.** bZip transcription factor binding motifs and their locations on the consensus sequence of putative *dab* integration sites of *N. navis-varingica* K0620, *P. multiseries* CNS00149, *P. pungens* CNS00055, and *P. delicatissima* CNS00130. Image produced by MEME Suite webpage (https://meme-suite.org).

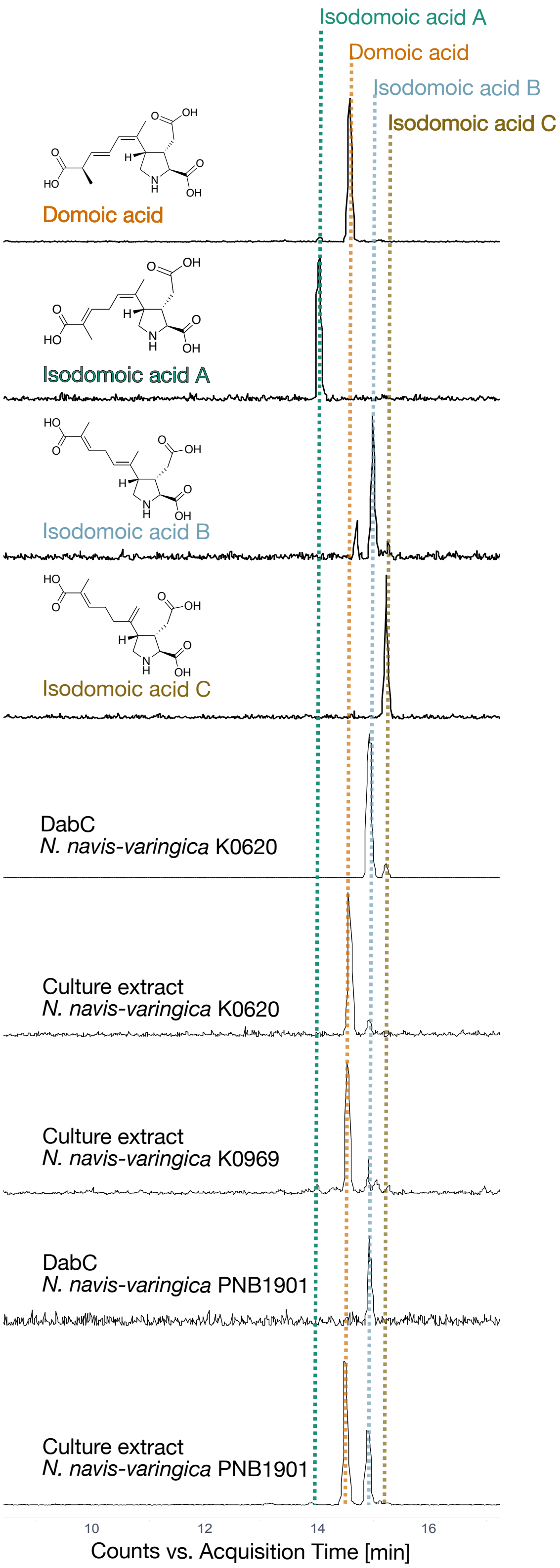


**Figure S12.** Culture extracts and DabC assays compared with synthetic standards. Positive ion mode LC-MS extracted ion chromatogram profiles for anticipated domoic acid and related isomer products (*m/z* 312.1±1.0).

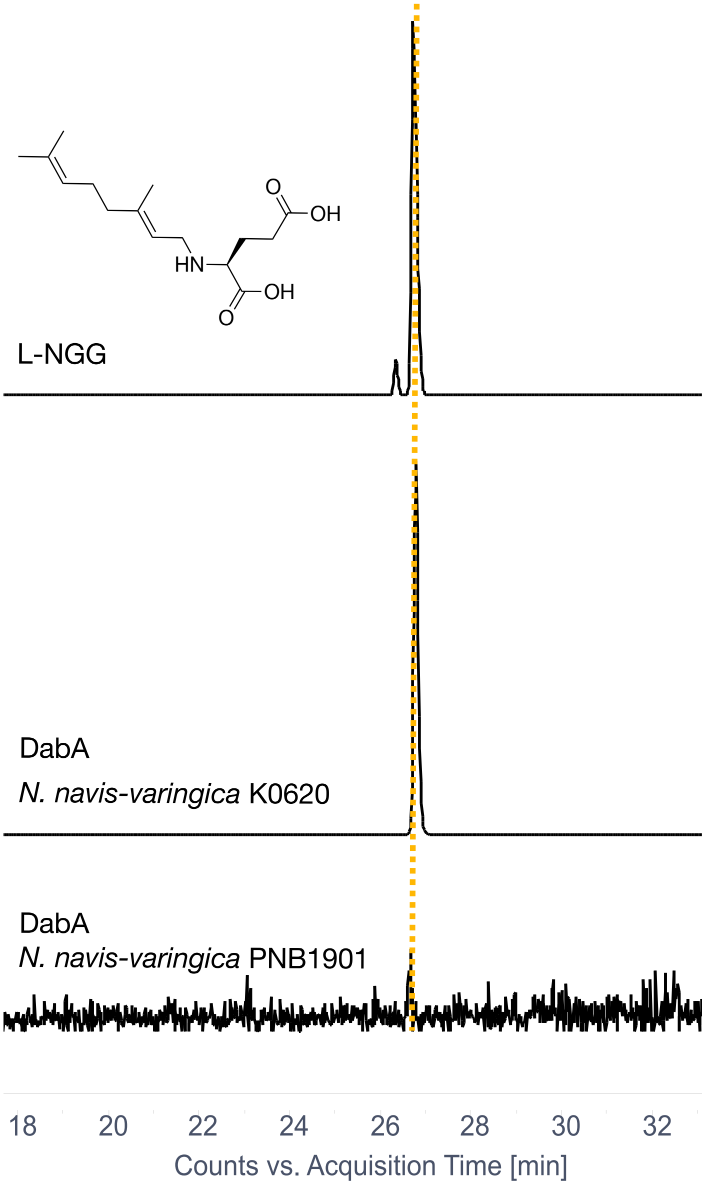


**Figure S13.** Positive ion mode LC-MS extracted ion chromatogram profiles for L-NGG (*m/z* 280.2 ± 1.0) from synthetic standard and DabA assays.

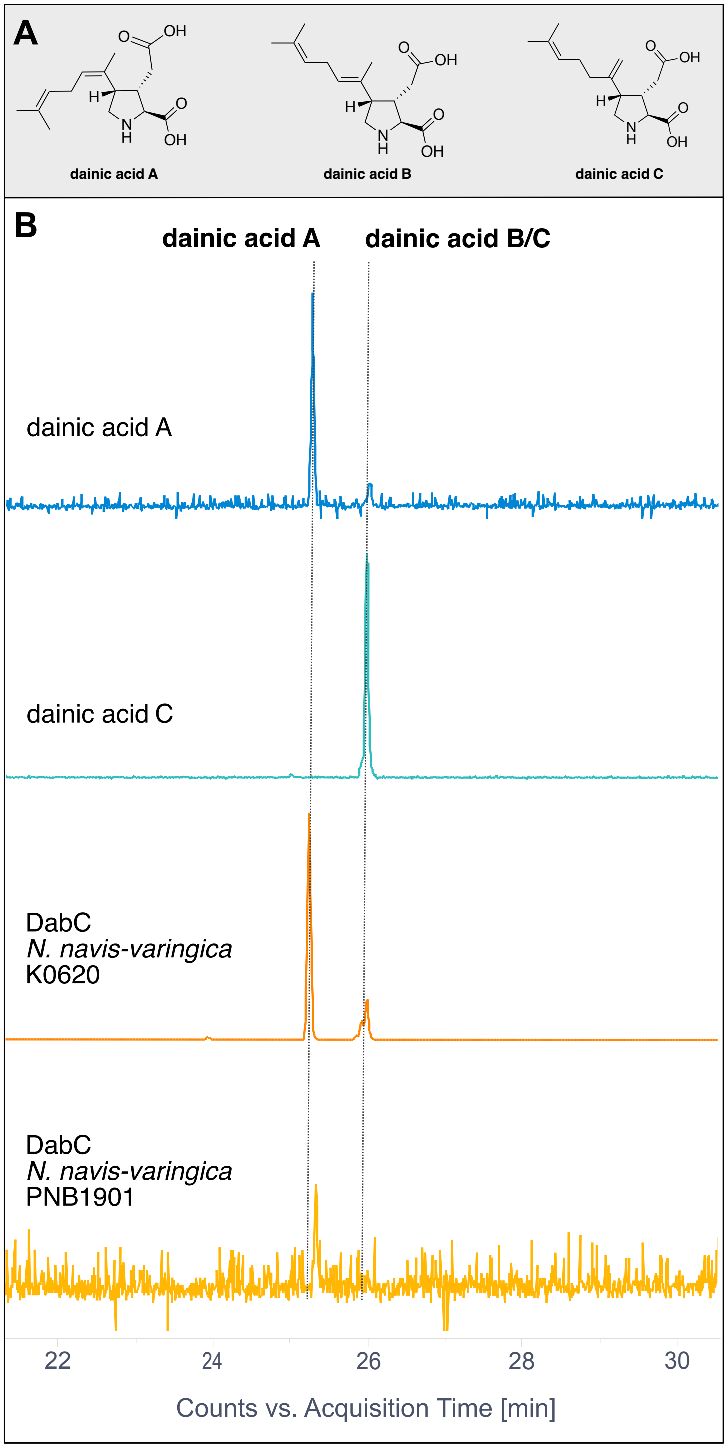


**Figure S14.** (A) Dainic acid isomers. (B) Positive ion mode LC-MS extracted ion chromatogram profiles for dainic acid isomers compared with synthetic standards (*m/z* 282.2±1.0). Dainic acids B and C have been shown to co-elute using our LC-MS methods.

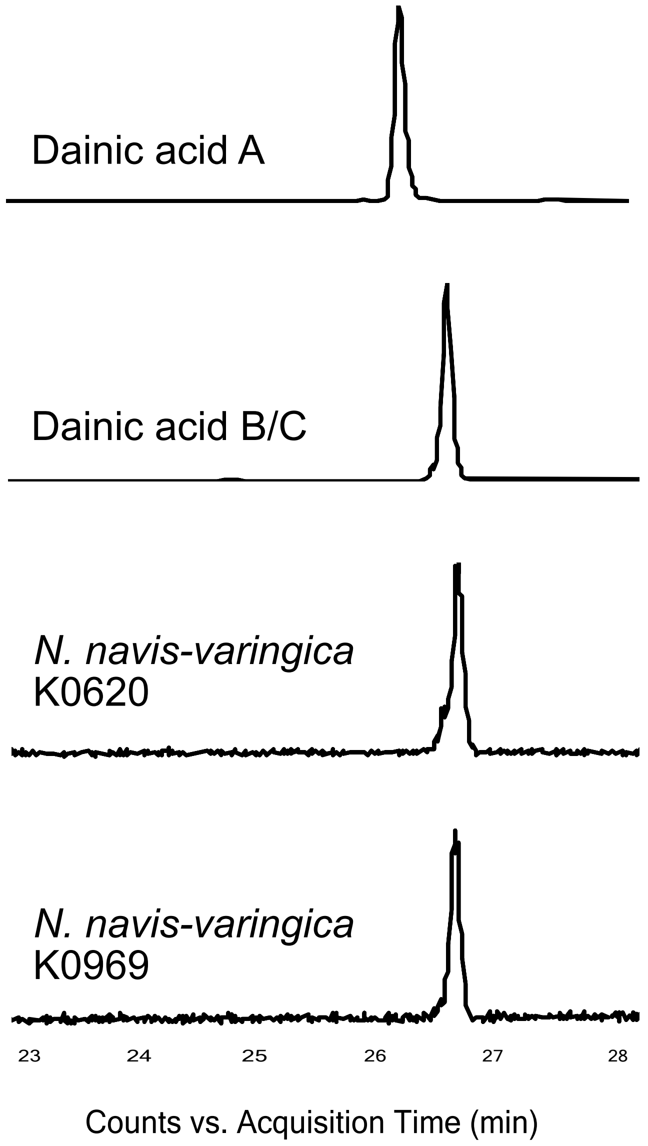


**Figure S15.** Dainic acid in culture extracts. Negative ion mode LC-MS extracted ion chromatogram profiles for dainic acid isomers compared with synthetic standards (*m/z* 280.2±0.1). Dainic acids B and C have been shown to co-elute using our LCMS methods.

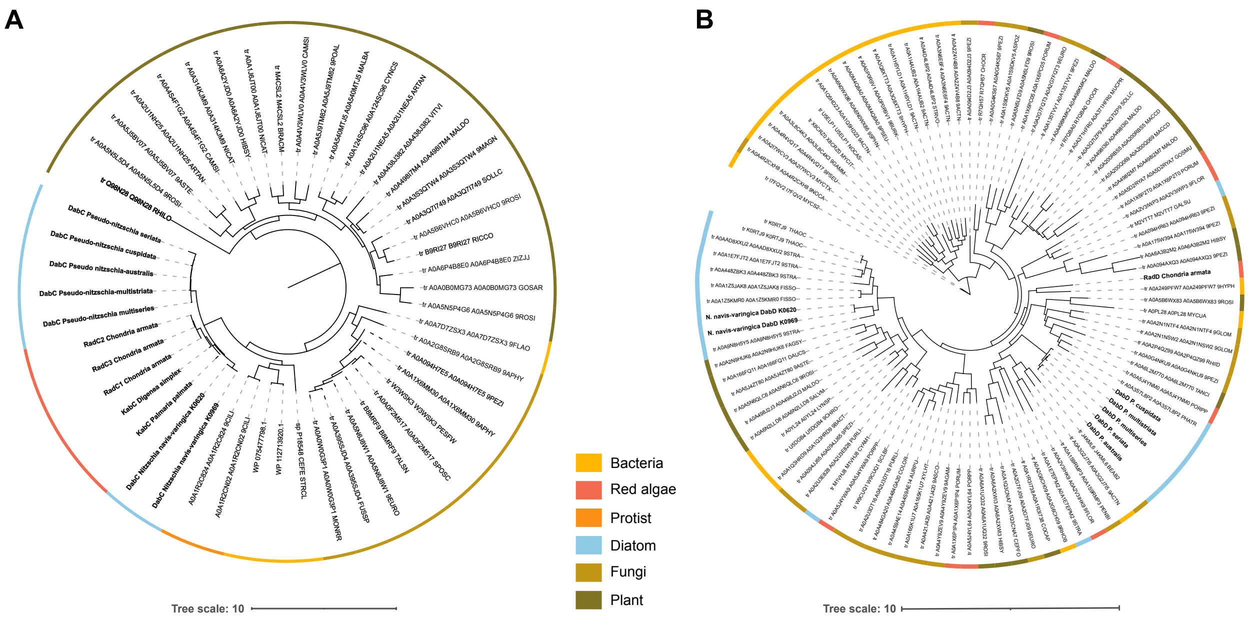


**Figure S16. Phylogenetic analysis of (A) kainoid synthase and (B) DabD CYP450 enzymes.** Maximum likelihood trees were constructed using Kalign and iTOL (EMBL). Kainoid synthase enzymes form a distinct branch, while *N. navis-varingica* DabD clusters with other diatom CYP450 sequences.

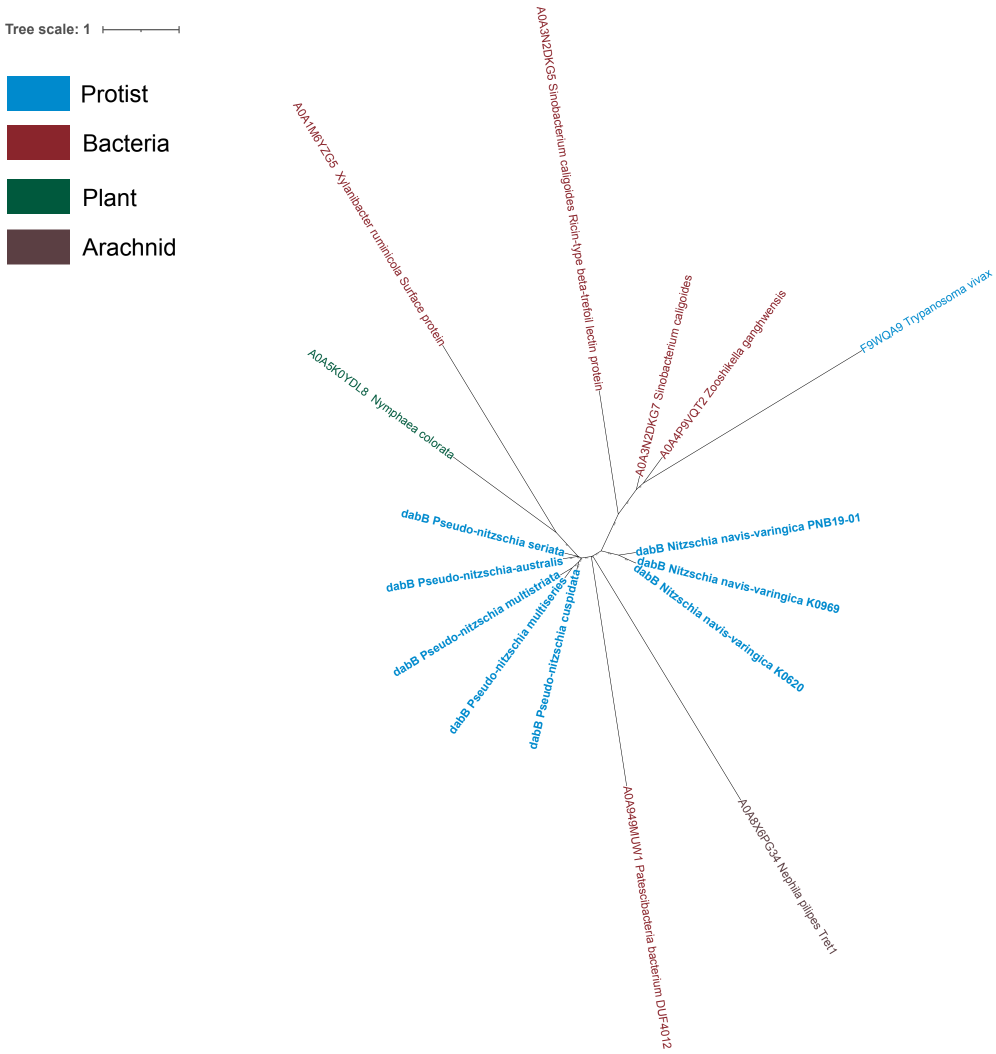


**Figure S17.** Unrooted phylogenetic analysis of DabB amino acid sequences.

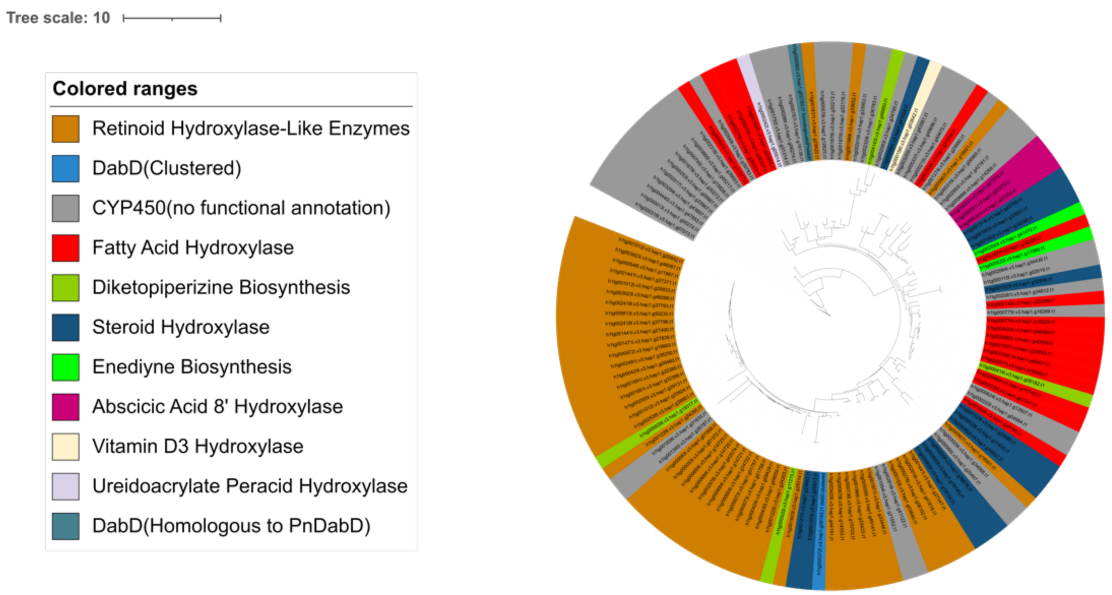


**Figure S18.** *Nitzschia navis-varingica* K0620 Cytochrome P450 phylogenetic tree. CYP function was annotated based on KEGG Orthology (KO).

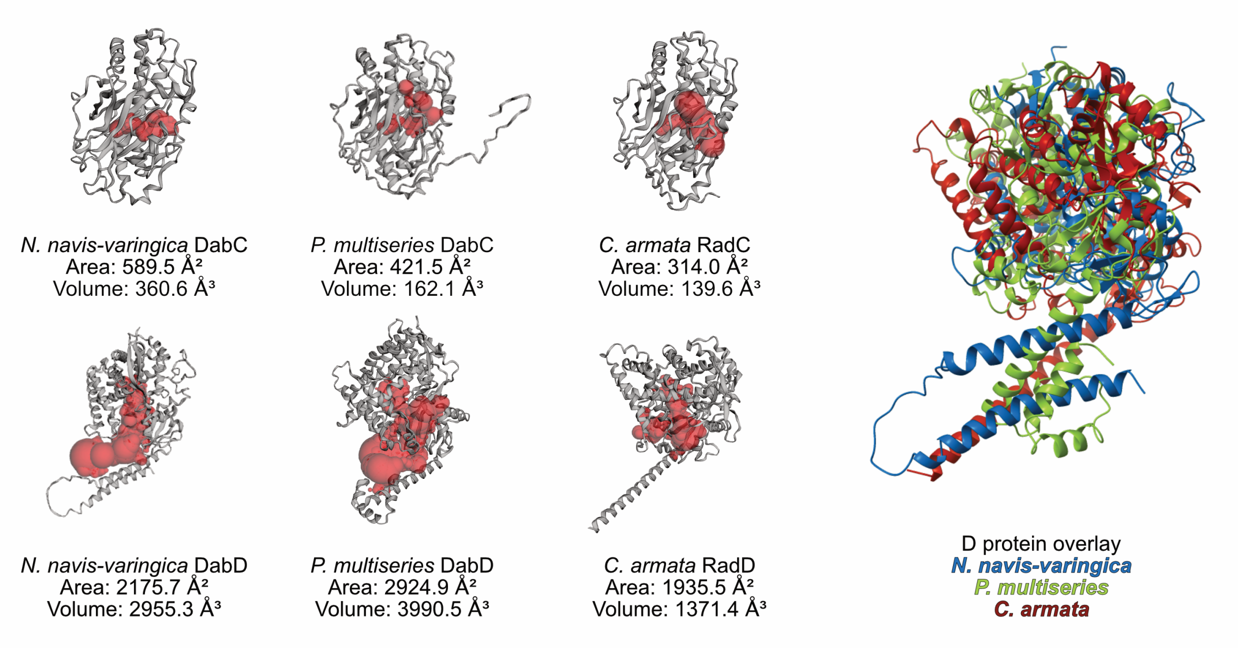


**Figure S19.** AlphaFold2 and CASTpFOLD output of structural models and modeled pocket volumes for *N. navis-varingica* K0620 DabC1, *P. multiseries* DabC, *C. armata* RadC1. Enzyme binding pockets highlighted in red, measurements of pocket area and volume listed below each model label. Images generated by CASTpFOLD webserver (https://cfold.bme.uic.edu/castpfold/). D-type CYP450 structures are overlaid for aided visualization, with *N. navis-varingica* DabC in blue, *P. multiseries* DabC in green, *C. arm*ata RadC1 in red. Image generated in ChimeraX (v1.1).
